# Sepsis induces long-term metabolic and mitochondrial muscle stem cell dysfunction amenable by mesenchymal stem cell therapy

**DOI:** 10.1038/ncomms10145

**Published:** 2015-12-15

**Authors:** P. Rocheteau, L. Chatre, D. Briand, M. Mebarki, G. Jouvion, J. Bardon, C. Crochemore, P. Serrani, P. P. Lecci, M. Latil, B. Matot, P. G. Carlier, N. Latronico, C. Huchet, A. Lafoux, T. Sharshar, M. Ricchetti, F. Chrétien

**Affiliations:** 1 Infection and Epidemiology Department, Institut Pasteur Human Histopathology and Animal Models Unit, 75724 cedex15, Paris, France; 2 Department of Developmental and Stem Cell Biology, Institut Pasteur, Stem Cells and Development, 75724 cedex15, Paris, France; 3 Team Stability of Nuclear and Mitochondrial DNA, CNRS UMR 3525, 75724 cedex15, Paris, France; 4 NMR Laboratory, Institute of Myology, Paris 75013, France; 5 CEA, I2BM, MIRCen, NMR Laboratory, Paris 75013, France; 6 Anesthesia and Reanimation Department, Department of Surgery, University of Brescia, Brescia 25121, Italy; 7 INSERM UMR1087/ CNRS UMR6291, Institut du Thorax, Therassay, Université de Nantes, Faculté des Sciences et des Techniques, F44322 Nantes 44000, France; 8 Service de réanimation médico-chirurgicale adulte, Hôpital Raymond Poincaré, Garches 92380, France; 9 Université Versailles Saint Quentin, Versailles 78000, France; 10 TRIGGERSEP, F-CRIN Network, Versailles 78000, France; 11 Laboratoire de Neuropathologie, Centre Hospitalier Sainte Anne, Paris 75014, France; 12 Paris Descartes University, Sorbonne Paris Cité, Paris 75006, France

## Abstract

Sepsis, or systemic inflammatory response syndrome, is the major cause of critical illness resulting in admission to intensive care units. Sepsis is caused by severe infection and is associated with mortality in 60% of cases. Morbidity due to sepsis is complicated by neuromyopathy, and patients face long-term disability due to muscle weakness, energetic dysfunction, proteolysis and muscle wasting. These processes are triggered by pro-inflammatory cytokines and metabolic imbalances and are aggravated by malnutrition and drugs. Skeletal muscle regeneration depends on stem (satellite) cells. Herein we show that mitochondrial and metabolic alterations underlie the sepsis-induced long-term impairment of satellite cells and lead to inefficient muscle regeneration. Engrafting mesenchymal stem cells improves the septic status by decreasing cytokine levels, restoring mitochondrial and metabolic function in satellite cells, and improving muscle strength. These findings indicate that sepsis affects quiescent muscle stem cells and that mesenchymal stem cells might act as a preventive therapeutic approach for sepsis-related morbidity.

## Abstract (web summary)

Sepsis patients often develop muscle atrophy that can last for years. Here the authors show in a mouse model that sepsis causes long-term impairment of the satellite cells, affecting mitochondrial function and energy metabolism, and that injection of mesenchymal stem cells restores satellite cell metabolism and muscle regeneration.

## Graphical abstract

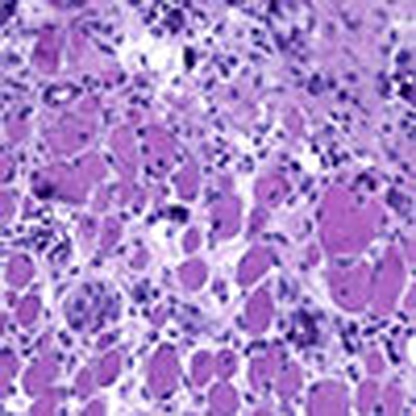

Sepsis is defined as an infection that causes an uncontrolled systemic inflammatory response that leads to vascular leakage, tissue damage and multiorgan failure. In many cases, sepsis results in swift death, and current treatments are not very effective^1^. Patients who survive frequently suffer from muscle wasting^2^,^3^,^4^. In the early phases of sepsis, catabolism of skeletal muscle can be beneficial because it provides glutamine to gut mucosa^5^ and to the immune system^6^, and supports gluconeogenesis and acute phase protein synthesis in the liver by providing amino acids^7^. However, if this catabolic activity persists, it provokes muscle loss and becomes detrimental^8^. This is especially true when respiratory muscles are targeted^9^. Indeed, continued loss of muscle proteins, particularly myofibrillar proteins^10^,^11^, results in muscle atrophy and weakness, which have significant clinical consequences. In survivors of critical illness, this physical disability can last for 5 years^12^,^13^.

Normally, skeletal muscle is capable of remarkable regeneration in response to injury or trauma, a property conferred by the presence of muscle stem cells, satellite cells (SCs)^14^,^15^,^16^. Although it is known that after sepsis the imbalance between anabolism and catabolism leads to muscle wasting, the mechanisms that cause the failure of muscle regeneration after extended periods of time are still not clear. To address this issue, we focus on muscle regeneration and SC function following septic shock. We show long-lasting mitochondrial and metabolic alterations in SC after sepsis, which are associated with inefficient muscle regeneration. We also show that these alterations, as well as high cytokine levels, are reverted by engrafting mesenchymal stem cells, resulting in improved septic state and increased muscle strength. These findings reveal that quiescent muscle stem cells are affected by sepsis and that mesenchymal stem cells may have use in preventive therapeutic approaches.

## Results

### Muscle is not able to regenerate after sepsis

**Figure 1:**
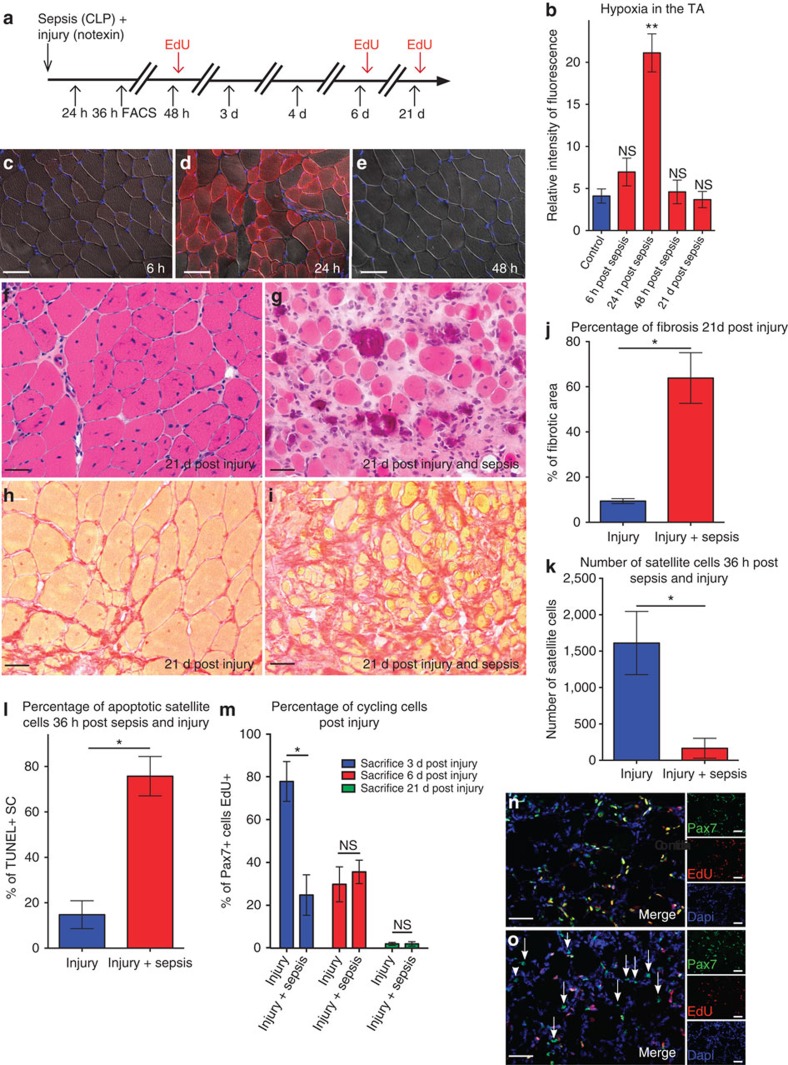
Muscle regeneration is impaired during sepsis. (**a**) Schematic representation of sacrifice post CLP and injury. (**b**) Intensity of fluorescence measured by microscopy and quantified using ImageJ of hypoxyprobe at different time points post CLP. (**c**–**e**) Hypoxyprobe immunostaining of cryosectioned TA muscle at 6, 24 and 48 h post induction of sepsis, respectively. Scale bar, 200 μm. Haematoxylin and eosin staining of a TA 21 days (d) post injury (**f**) and 21 days post injury and CLP (**g**) in 8-week-old mice. Scale bar, 100 μm. Sirius Red staining of TA muscle 21 days post injury (**h**) or 21 days post sepsis and injury (**i**). Scale bar, 100 μm. (**j**) Percentage of fibrotic area measured using the threshold method with ImageJ in septic injured mice versus healthy injured mice. (**k**) Absolute number of satellite cells (counted by FACS) isolated 36 h post injury in the TA muscle of septic (CLP) *Tg:Pax7nGFP* transgenic mice (*n*=5). (**l**) Percentage of TUNEL-positive cells 36 h post CLP and injury, *n*=5 *TgPax7nGFP* mice killed and satellite cells FACS cell sorted per condition. (**m**–**o**) Percentage of cycling SC at different time points post injury and sepsis. (**m**) Percentage of EdU-positive SC in injured (controls), and injured and septic animals at different time points post injury. (**n**) Representative image of double Pax7/EdU staining 3 days post injury (regeneration in control mice). (**o**) Representative picture of double Pax7/EdU staining 3 days post injury and sepsis (arrows point single-labelled satellite cells, that is, non-cycling SC). On the right (lower magnification) individual staining is shown. For all time points mice were between 8 and 12 weeks old, and *n*=8 animals were analysed. Data are represented as mean±s.d. *P<0.05; **P<0.01 (Mann–Whitney test). NS, not significant.

Sepsis was induced in mice by caecal ligature and puncture (CLP), which generates an exacerbated immune response and simulates clinically relevant human conditions^17^. Twenty-four hours post CLP, we observed severe but transitory hypoxia in the tibialis anterior (TA) muscle of septic mice (Fig. 1a–e), despite normal histology (Supplementary Fig. 1a–m). The hypoxic condition could not be ascribed only to reduced perfusion. Indeed, functional magnetic nuclear resonance (MNR) analysis revealed large variability in the response to perfusion after sepsis (11 ml per min per 100 g±12, mean±s.d., see Supplementary Table 1), whereas hypoxia was similar in all septic mice. The muscle regenerative capacity of septic mice was assessed after injury of the TA muscle with notexin at the time of CLP. We note that, unlike control mice, muscle regeneration was compromised in septic mice as revealed by: (i) marked anisocytosis and a high proportion of small atrophic myofibers (21 days after injury, fibre size 123±35 μm^2^ post injury versus 45±40 μm^2^ post injury and sepsis; and 104±64 fibres per mm^2^ post injury versus of 35±24 fibres per mm^2^ post injury and sepsis); (ii) endomysial fibrosis, representing 64±11% of the total muscle section surface area; (iii) persistence of chronic endomysial inflammation; and (iv) calcification of necrotic myofibers accompanied by multinucleated giant cells (Fig. 1f–j). To support these histological observations, we assessed the levels of creatine kinase, a marker of necrosis. Twenty-one days post injury, creatine kinase returned close to basal levels in the injured control mice, whereas it remained high in the septic mice (Supplementary Fig. 1n). These effects persisted at later time points, and regeneration was defective whether injury was performed at the same time or 4 days to 3 months post CLP (Supplementary Fig. 2a–g).

### Satellite cells are impaired after sepsis

**Figure 2:**
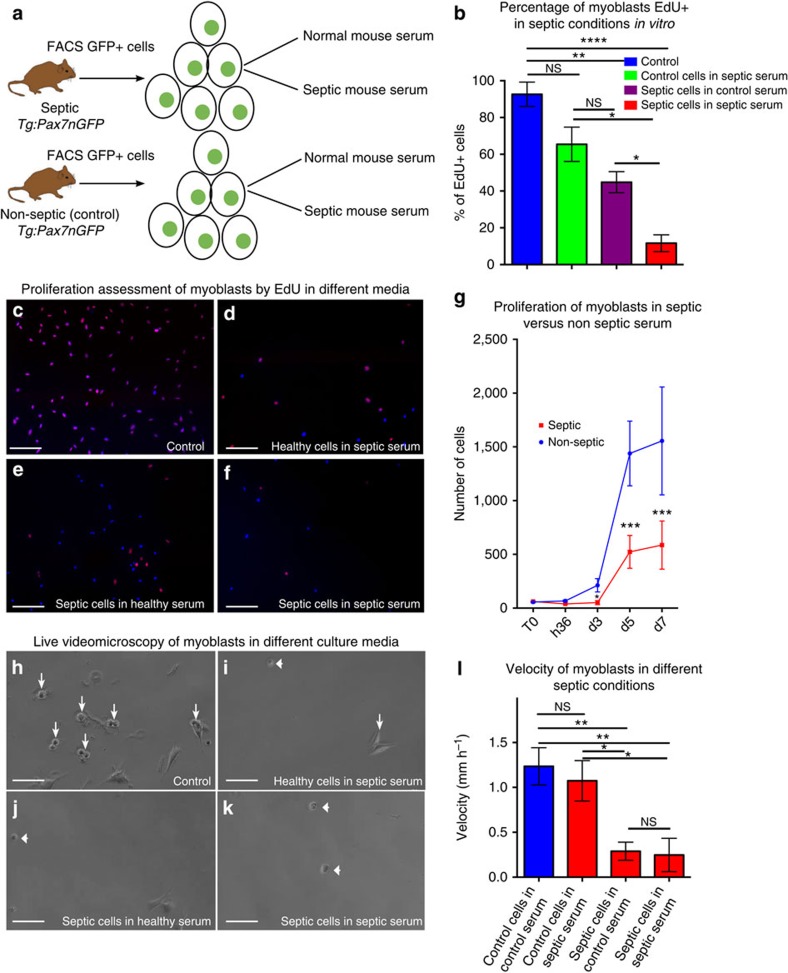
Muscle satellite cell division is impaired in the presence of septic serum. (**a**) Schematic representation of the experimental approach for the *in vitro* test of septic conditions. (**b**–**f**) Percentage of cycling cells in different septic conditions *in vitro.* (**b**) Percentage of EdU+ myoblasts (cycling satellite cells) cultured in regular conditions (control healthy *Tg:Pax7nGFP* mice, control serum) or extracted from septic *Tg:Pax7nGFP* in normal serum (septic animal in control serum) or extracted from healthy *Tg:Pax7nGFP* in septic serum (control animal in septic serum) or extracted from septic *Tg:Pax7nGFP* in septic serum. (**c**–**f**) Representative picture of FACS cell-sorted satellite cells from *Tg:Pax7nGFP* mice and plated in the different conditions described above, labelled with EdU and fixed/labelled 3 days post plating. (**c**) Non-septic *Tg:Pax7nGFP* in non-septic mouse serum. (**d**) Healthy *Tg:Pax7nGFP* in septic serum. (**e**) Septic *Tg:Pax7nGFP* in non-septic serum. (**f**) Septic *Tg:Pax7nGFP* in septic serum. (**g**) Cells from healthy (non-septic) and septic *Tg:Pax7nGFP* mice were FACS cell sorted and plated at 100 cells per well density. Clonal analysis of the proliferation through time (from T0 to 7 days post plating) was performed using Opera system (automatic clonal cell count). (**h**–**l**) Live video microscopy of all conditions previously mentioned (see **a**). (**h**) Control (that is, healthy cells in non-septic mouse serum), (**i**) healthy cells in septic serum, (**j**) septic cells in non-septic serum and (**k**) septic cells in septic serum. (**l**) Velocity of cells measured in μm h^−1^ in all four conditions. *Tg:Pax7nGFP* mice were between 8 and 12 weeks old, *n*=4. Data are represented as mean±s.d. *P<0.05; **P<0.01; ***P<0.001; ****P<0.0001 (Mann–Whitney test). GFP, green fluorescent protein; NS, not significant.

We then focused on SCs, which play a central role in muscle homeostasis and regeneration^14^,^15^,^16^. Using *Tg*:*Pax7nGFP* transgenic mice to isolate SCs by fluorescence-activated cell sorting (FACS)^18^, we observed a marked decrease (89%) in SC number 36 h post injury (Fig. 1k) due to apoptosis (76±9% TUNEL-positive SCs, Fig. 1l, and 73±11% cleaved caspase-3 SC, Supplementary Fig. 2h–j). These data were confirmed using *Pax7*^*LacZ/+*^mice, which displayed loss of 80% of SCs after sepsis and injury (Supplementary Fig. 2k,l). Of the SCs surviving sepsis, 24.8±9.4% were actively cycling 3 days post injury compared with 77.8±9.3% in controls (Fig. 1m–o). The long-term division potential of SCs was also affected: only 35.5±11% of cells were actively cycling over 3 days (Supplementary Fig. 2m–o). These data were further confirmed by culturing SCs *in vitro* from control and septic *Tg:Pax7nGFP* mice in normal as well as in septic mouse serum (Fig. 2a–g). The septic serum was extracted from the blood of septic mice 24 h post CLP, and healthy serum was obtained from non-treated mouse blood. SC division was impaired when cultured in a septic environment or when originating from septic mice suggesting that not only extrinsic (Fig. 2b,d,g) but also intrinsic factors (Fig. 2b,e,g) affected SC division after sepsis. Moreover, except for controls in normal serum, SCs were unable to differentiate into myofibers in all other conditions (Fig. 2d–f). SC velocity also markedly decreased, as measured by live video microscopy (Fig. 2h–l and Supplementary Movies 1–4). The expression of MyoD, one of the earliest myogenic commitment markers^19^,^20^, was impaired in the septic condition. Indeed, after 24 h, 91±7% of SCs expressed MyoD in injured mice versus 34±5% of SCs surviving injury and sepsis (Supplementary Fig. 2p). These results were confirmed 4 days after the induction of sepsis and injury. The myogenic factor myogenin (MyoG), a marker of differentiation^21^, was also expressed at lower levels in septic conditions than in controls. *In vivo*, 8-fold and 49-fold fewer SCs expressed MyoG in septic mice 4 days and 7 days, respectively, post injury and sepsis compared with non-septic injured mice (Supplementary Fig. 2q). These results were confirmed *in vitro.* Indeed, 4 days post plating, 4±1.7% of FACS-sorted SCs in septic serum were positive for MyoG versus 27±7.5% of SCs in non-septic serum. At 7 days post plating, 8±1.5% of the few surviving SCs were MyoG positive in septic serum versus 70±4% in controls (Supplementary Fig. 2r).

In agreement with our *in vivo* observations, these data show that only 11% of SCs resist sepsis; most of these cells have compromised activation and proliferation as well as impaired expression of myogenic markers.

### Altered mitochondrial activity in impaired SCs after sepsis

Analysis of muscle bioenergetics, assessed by ^31^P-NMR spectroscopy, revealed significant metabolic impairments during the early stages of sepsis (lower muscle pH and PCr/ATPg, reflecting abnormal energy metabolism in septic muscle tissue compared with controls; Supplementary Table 1). To better understand the functional effects of sepsis on SCs and the pathophysiologic mechanisms behind alterations in muscle metabolism, we assessed mitochondrial parameters in SCs during sepsis.

**Figure 3:**
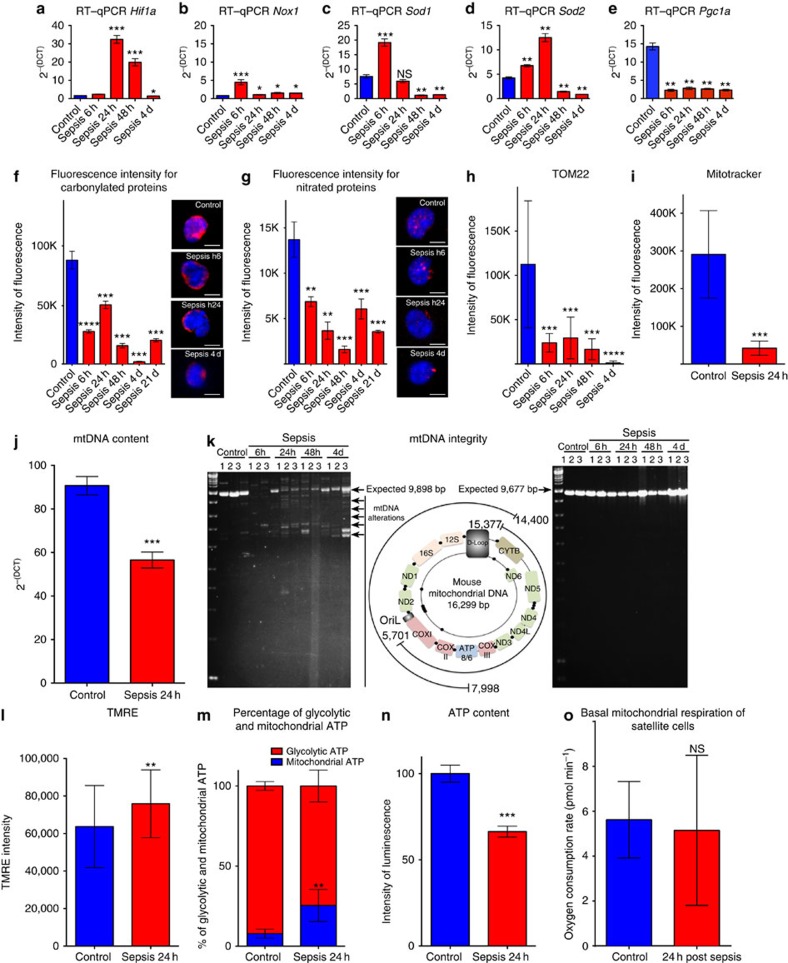
On sepsis satellite cells display altered but hyperactive mitochondria. (**a**–**e**) Quantification by RT–qPCR of nuclear genes in the time course of sepsis in the control (no injury no CLP) at 6 h, 24 h, 48 h and 4 days (d) post induction of sepsis; *n*=6 per time point; (**a**) expression of hypoxia-inducible factor1 (*Hif1a*); (**b**) NADPH oxidase 1 (*Nox1*); (**c**,**d**) expression of antioxidant enzymes *Sod1* and *Sod2*; (**e**) peroxisome proliferator-activated receptor coactivator 1 (*PGC1a*). (**f**,**g**) Quantification of immunofluorescence intensity by confocal microscopy image acquisition and ImageJ analysis. The levels of carbonylated (antibody anti-dinitrophenyl) and nitrated (antibody anti-nitrotyrosine) proteins (resulting essentially from reactive oxidative and nitrosative species, respectively) were measured in controls, 6 h, 24 h, 48 h and 4 days post induction of sepsis. Representative immunostaining (three-dimensional-reconstructed cells) are displayed. Scale bar, 5 μm. (**h**) Confocal microscopy immunofluorescence intensity quantification of TOM22 (mitochondrial outer membrane protein) and (**i**) confocal microscopy intensity of MitoTracker Deep Red staining probe in control (healthy) and septic mice. Once acquired by confocal microscopy the images were analysed using the mean grey value ImageJ plugin. (**j**) Mitochondrial DNA content (mtDNA) in control and septic SC. (**k**) Agarose gel electrophoresis of long PCR amplifications on sorted SC mtDNA in controls and septic *Tg:Pax7nGFP* 6 h, 24 h, 48 h and 4 days post sepsis showing mtDNA size alterations. Left panel, amplification of a fragment of expected size (9,898 bp) from positions 7,998–16,299/1 (conventional end/origin of circular mtDNA) and 1–14,400 of the mtDNA; the image on this panel is intentionally highly contrasted to underscore multiple bands, and also the low intensity of the expected band (9,898 bp) in sepsis samples compared with controls; centre panel, schematic representation of genes and regulatory regions in the human mtDNA, with the coordinates of PCR-amplified regions; right panel, amplification of a fragment of expected size 9,677 bp from positions 15,377 to 5,701 of the mtDNA. Note that size alterations in cells from septic mice are present in one (left panel) but not the other PCR-amplified fragment. (**l**) Tetramethylrhodamine ethylamine (TMRE; mitochondrial membrane potential) levels. (**m**) Relative percentage of glycolytic and mitochondrial (OXPHOS) ATP in controls (no injury no CLP) and 24 h post sepsis. (**n**) Percentage of ATP relative to control in controls (*n*=6) and septic *Tg:Pax7nGFP* at 24 h (*n*=6). (**o**) Basal mitochondrial respiration of SC in non-septic (control) and 24 h post sepsis evaluated with Seahorse XFe96 analyser. Unless specified data are represented as mean±s.d. *P<0.05; **P<0.01; ***P<0.001; ****P<0.0001; NS, not significant, compared with the respective control (Mann–Whitney test).

We observed transitory stimulation of the expression of hypoxia-inducible factor-1 alpha (*Hif1a*)^22^ from 24 to 48 h post sepsis (Fig. 3a), in accordance with the hypoxia observed in the TA muscle at 24 h (see above, Fig. 1b). *Hif1a* is induced by reactive oxygen species (ROS)^23^, and accordingly, we observed ROS levels peaking at 24 h post sepsis (Supplementary Fig. 3a). Six hours after induction of sepsis, NADPH oxidase 1 (*Nox1*)^24^ and antioxidant defence factors, specifically cytosolic *Sod1* and mitochondrial *Sod2* superoxide dismutase factors^25^, were upregulated in SCs (Fig. 3b–d), suggesting an active response to oxidative stress. Superoxide dismutase (SOD) activity increased with time post sepsis, even with low RNA levels (Supplementary Fig. 3b). Consistent with a strong antioxidant defence, we observed a rapid decrease in carbonylated and nitrated protein levels^26^ in SCs (Fig. 3f,g) that persisted for 21 days post sepsis (this was also the case for ROS, Supplementary Fig. 3a), indicating reduced nitroso-redox stress^17^. The reduction in protein nitration was specific to satellite cells because myofibers displayed increased protein nitration 24 h post sepsis (Supplementary Fig. 3c,d,g). Conversely, protein carbonylation was reduced in SCs as well as in myofibers (Supplementary Fig. 3e,f,h). Strikingly, the expression of *Pgc1a*, a master regulator of ROS detoxification, gluconeogenesis, mitochondrial biogenesis, metabolism and muscle wasting^27^, markedly and persistently decreased in SCs after sepsis (Fig. 3e). Accordingly, we observed a remarkable and persistent reduction of the mitochondrial mass (≥70%), measured with MitoTracker Deep red and by assessing mitochondrial translocase TOM22 immunofluorescence (Fig. 3h,i)^28^,^29^. The decrease in mitochondrial mass was specific to SCs because after sepsis the mitochondria of myofibers displayed increased MitoTracker Deep Red and TOM22 signals (Supplementary Fig. 4a–e).

Mitochondrial activity strongly depends on mitochondrial DNA (mtDNA). SCs lost 40% of their mtDNA by 24 h post sepsis (Fig. 3j). Intriguingly, the reduction in mtDNA was uncoupled to the initiation of mtDNA replication. Global mitochondrial transcript content (mTRANS probe) and initiation of mtDNA replication (mREP probe) were assessed *in situ* and at single-cell resolution^28^ (Supplementary Fig. 5a,b), and transcripts of mitochondrial genes *CytB* and *16S* rRNA were assessed by real-time quantitative PCR (RT–qPCR) (Supplementary Fig. 5c,d)^28^,^29^. Moreover, we detected a marked accumulation of mtDNA size alterations from 6 h to 4 days post sepsis (with alterations located in an ≈6-kbp region of the mtDNA), supporting the notion of persistent mitochondrial dysfunction after sepsis (Fig. 3k). At 24 h post sepsis, residual mitochondria were hyperpolarized (Fig. 3l). In addition, although quiescence favours glycolysis^30^, we detected a slight increase of oxidative phosphorylation (OXPHOS) in SCs during sepsis, despite an overall decrease in ATP levels (Fig. 3m,n), reflecting the hyperactivity of mitochondria surviving sepsis in SCs, but no increase in O_2_ consumption rate (Fig. 3o). Bioenergetics analysis performed with Seahorse technology revealed that the rates of maximal respiration, proton leak, spare respiratory capacity and coupling efficiency were comparable in septic SCs and controls (Supplementary Fig. 4f–l). Altogether, sepsis had rapid, strong and persistent effects on mitochondrial mass, mtDNA content and mtDNA integrity. Nevertheless, despite an unfavourable environment, residual mitochondria appeared hyper-replicative, hyper-transcription active and hyperpolarized, leading to a metabolic shift towards OXPHOS. This altered mitochondrial activity and metabolic unbalance could explain the impaired ability of SCs to regenerate muscle.

### Mesenchymal stem cells restore mitochondrial function

**Figure 4:**
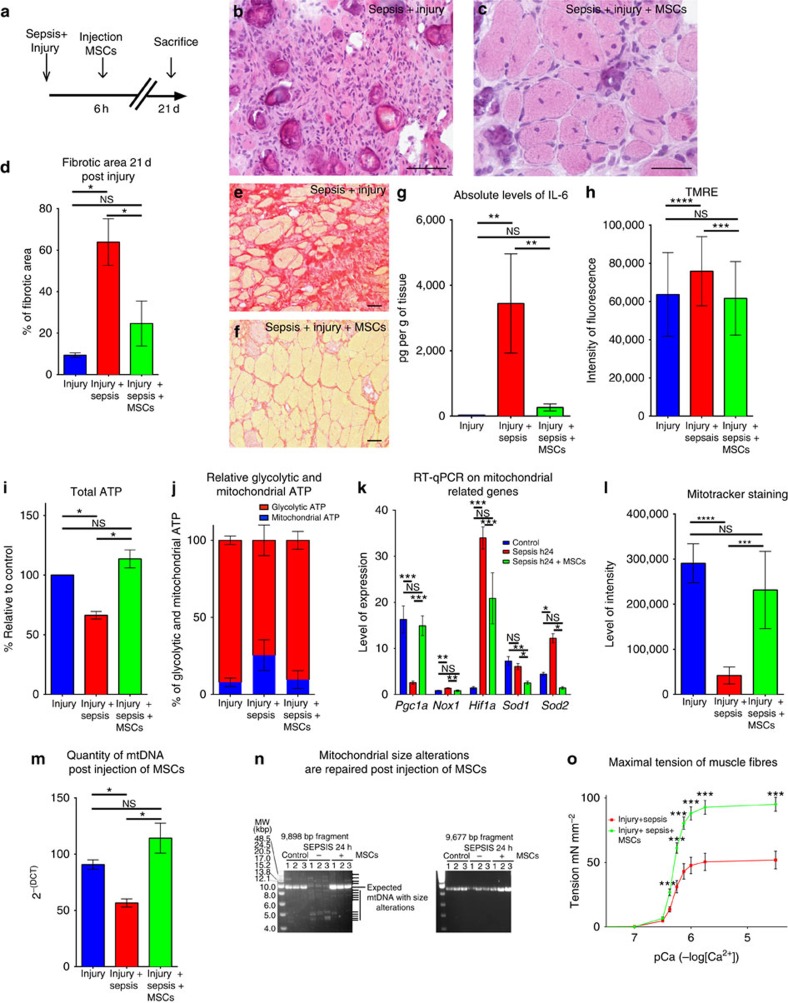
Mesenchymal stem cells improve muscle regeneration by decreasing septic state and restoring affected mitochondrial parameters in satellite cells. (**a**) Schematic representation of timing of sacrifice, post-caecal ligature puncture and injury (with notexin) plus MSCs grafting. (**b**) Haematoxylin and eosin staining of the TA 21 days (d) post sepsis and injury (*n*=16). Scale bar, 100 μm. (**c**) Haematoxylin and eosin of TA 21 days post sepsis and injury with injection 6 h post injury of mesenchymal stem cells (*n*=16). Scale bar, 100 μm. (**d**–**f**) Fractions of fibrotic area. (**d**) Fibrotic area of the muscle tissue after regeneration (21 days post injury) in control, septic and septic injected with MSC conditions. (**e**) Sirius Red staining of muscle tissue of septic mice 21 days post injury (*n*=16) Scale bar, 100 μm. (**f**) Sirius Red staining of muscle tissue 21 days post injury of septic mice treated with MSC injection (*n*=16). Scale bar, 100 μm. (**g**) Example of Luminex on interleukin-6 (IL-6) representing the level of protein expression in pg g^−1^ of muscle tissue. Control are non-injured mice (*n*=4), septic *Tg:Pax7nGFP* at 24 h (*n*=4) and septic *Tg:Pax7nGFP* injected with MSCs (*n*=4). (**h**) TMRE label in injured (*n*=3); injured, septic *TgPax7nGFP* (*n*=3); and injured, septic, MSC-injected *TgPax7nGFP* (*n*=3) at 24 h. (**i**) ATP content relative to control (*n*=6), septic *TgPax7nGFP* (*n*=6) and septic *TgPax7nGFP* injected with MSCs (*n*=6) at 24 h. (**j**) Relative glycolytic and mitochondrial ATP content in controls (no injury no CLP), 24 h post sepsis, and septic *Tg:Pax7nGFP* injected with MSCs. (**k**) Quantification by RT–qPCR of *PGC1a*, *Nox1*, *Hif1a*, *Sod1* and *Sod2* expression. (**l**) Relative level of intensity of MitoTracker Deep Red in controls, septic *Tg:Pax7nGFP* and septic *Tg:Pax7nGFP* injected with MSCs at 24 h (*n*=6). (**m**) Quantification of mtDNA in FACS cell-sorted satellite cells in injured, injured and septic, and injured, septic and MSC-injected *Tg:Pax7nGFP* mice. (**n**) Agarose gel electrophoresis of long PCR amplification of mtDNA in SCs from controls, septic and septic mice injected with MSCs showing mtDNA alterations only in septic mice (left panel). The coordinates of the amplified fragments are shown in Fig. 3k. MSCs rescue normal mitochondrial genome size after sepsis. (**o**) Maximal tension of muscle fibres of septic and injured (*n*=6) compared with septic, injured and MSC-injected C57Bl/6 mice (*n*=7) according to the Ca^2+^ concentration. Data are represented as mean±s.d. *P<0.05; **P<0.01; ***P<0.001; ****P<0.0001; not significant, compared with the respective control (Mann–Whitney test).

Exogenous administration of purified mesenchymal stem cells (MSCs) has been shown to have a protective effect against sepsis in different organs^31^,^32^,^33^,^34^. To investigate if MSCs had a protective effect on satellite cells, we sorted MSCs from non-septic C57Bl/6 bone marrow by FACS (CD45−, Ter119−, PDGFR1a+ and Sca1+)^35^ and injected 300,000 cells intramuscularly in 15 μl of 0.9% NaCl, 6 h after induction of sepsis and injury (Fig. 4a). Under these conditions and at that time, SCs were already impaired (see above, Fig. 3a–h,k). Muscle regeneration, evaluated histologically by examining newly formed myofibers 21 days post injury and sepsis, was more efficient in the presence of MSCs (104±64 fibres per mm^2^) compared with untreated septic and injured controls (35±24 fibres per mm^2^, *P*≤0.001), with larger fibres (45±40 μm^2^ post injury and sepsis versus 112±69 μm^2^ post injury, sepsis and MSC treatment, *P*≤0.01; Fig. 4b–c). Moreover, MSC-treated mice displayed less fibrosis and necrosis than controls (Fig. 4d–f and Supplementary Fig. 5e). The initial drop in leukocytes after CLP^36^ was restored 3 days after MSC injection (Supplementary Fig. 5f). The leukocyte count in the blood indicated the absence of infection 21 days after induction of sepsis (in survivors), either with or without injection of MSCs (Supplementary Fig. 5g). Moreover, the level of procalcitonin, an early indicator of sepsis, was closer to the level of the controls when mice were injected with MSCs (Supplementary Fig. 5h). We also observed a decrease in the levels of cytokines 24 h after injection of MSCs, as exemplified by interleukin-6 (Fig. 4g and Supplementary Fig. 6a). Furthermore, we report lower levels of cytokines in the septic TA injected with MSCs compared with the contralateral, non-injected TA (Supplementary Fig. 5i). When injured, non-injected TA muscles were unable to regenerate properly (42±15 fibres per mm^2^ (Supplementary Fig. 5j)).

On MSC injection, most mitochondrial parameters returned to normal in FACS-sorted SCs: that is, mitochondrial membrane potential (Fig. 4h), ATP levels (Fig. 4i), relative percentage of glycolytic and mitochondrial ATP (Fig. 4j), expression of mitochondrial biogenesis (*Pcg1a*) and antioxidant response (*Sod2*) factors (Fig. 4k), mitochondrial mass (Fig. 4l) and mtDNA content (Fig. 4m). Moreover, after injection of MSCs, the mtDNA alterations observed in SCs 24 h post induction of sepsis disappeared in favour of full-size mtDNA (Fig. 4n). Thus, fast recovery of mitochondrial status in septic SCs was subsequent to MSC treatment and linked to restored regeneration of muscle in septic mice.

MSCs have been shown to differentiate into multiple lineages^37^, including muscle^38^, depending on the surrounding environment^39^. After injecting MSCs, isolated as previously described, into double-transgenic *Tg:CAG-hPLAP::MLC3F-nlacZ-2E* septic injured mice (mice carrying both the human placental alkaline phosphatase gene that is expressed ubiquitously^39^ and LacZ that marks differentiated myonuclei^40^), we did not detect any PLAP-positive fibres, any PLAP-positive SCs or X-gal staining (Supplementary Fig. 6b–d). This indicates that the donor MSCs did not differentiate into muscle or SCs. To investigate whether MSC injection and subsequent improvement of muscle regeneration had functional consequences, contractile protein properties and force production were assessed using skinned fibre experiments. In fibres isolated from septic and injured TA muscle, the tension significantly increased from pCa 6.25 to pCa 4.5 after MSC injection (Fig. 4o). Thus, although the maximal Ca^2+^-activated tension increased by 91.4±10.2% (*n*=35 fibres sampled), we did not observe modifications in Ca^2+^ sensitivity or the Hill coefficient (Supplementary Fig. 6e,f), indicating a larger loss of contractile proteins in non-injected versus MSC-treated TA, and suggesting that the fibre type composition did not change after MSC treatment^41^,^42^. Furthermore, although injection was limited to the TA, we evaluated the recovery of the entire limb. The grip test performed 21 days after MSC injection, although globally nonsignificant, revealed that several septic mice injected with MSC displayed larger body force than non-injected mice (Supplementary Fig. 6g). Altogether, these data demonstrate that MSC treatment after sepsis induces more efficient muscle regeneration and has a functional impact by improving muscle fibre tension.

## Discussion

Critical illness myopathy is a major complication of sepsis^43^,^44^, which is one the main causes of admission and mortality in the intensive care unit (ICU). Critical illness myopathy affects up to 4% of septic patients and is independently associated with increased ICU and post-ICU mortality^12^,^44^, failure of weaning from mechanical ventilation and long-term disability^12^. The pathophysiology of critical illness myopathy is complex and multifactorial, involving mainly four mechanisms: (1) impairment of membrane excitability related to ion channel dysfunction; (2) energetics impairment related to mitochondrial dysfunction; (3) proteolysis, mainly by activation of the ubiquitins–proteasome complex; and (4) disturbance of intracellular calcium homeostasis. These pathophysiological processes then result in protein contractile dysfunction or loss, thereby accounting for muscle weakness and wasting. Notably, muscle wasting itself impairs and delays muscle recovery^8^. In addition, critical illness myopathy can be associated with axonal polyneuropathy that worsens and prolongs muscle dysfunction. These mechanisms are triggered by various factors related to either sepsis or to ICU management, including circulating pro-inflammatory cytokines, electrolyte and metabolic disturbances, endocrine imbalance between anabolic and catabolic hormones, myotoxic drugs (such as steroids) and unloading (that is, disuse). Disuse is a major therapeutic target for reducing muscle weakness and wasting. Indeed, various strategies are now recommended, including early discontinuation of sedation, weaning from mechanical ventilation and mobilization. Our findings might be related to disuse at least to some extent because sepsis markedly decreases mobility in the mice for the first few days. However, this disuse was not as intense and prolonged as that observed in septic patients, and the mice moved normally 5 days post CLP. Therefore, we think that disuse is unlikely to contribute to muscle weakness in our animal model, and thus, we have assessed muscle weakness but not muscle wasting in our model.

The muscle weakness, wasting and fatigue can persist for up to 5 years, with a marked impact on functional status and quality of life. This outcome suggests an impairment of muscle regeneration, which relies on SCs^14^,^15^,^16^. However, the hypothesis of SC dysfunction in critical illness myopathy has not been assessed to the best of our knowledge. In this study, we have addressed this hypothesis using a validated model of critical illness myopathy based on CLP and a validated model of injury^15^. We identified a novel mechanism for critical illness myopathy, that is, an early and long-lasting dysfunction of satellite cells that durably impairs muscle regeneration, replacing a fully functional muscle with a huge fibrotic area. Indeed, as early as 6 h post induction of sepsis, we observed a massive loss of proliferating/activated SCs. The remaining SCs displayed abnormal mitochondrial activity along with loss of mitochondrial mass and degraded mtDNA, but they also displayed hyper-replication-active, hyper-transcription-active and hyperpolarized organelles leading to an increase of OXPHOS. Although glycolysis remained prevalent, as for quiescent cells that are characterized by low metabolism^30^, septic SCs relied more extensively on mitochondria for ATP production than controls, in spite of markedly altered organelles. This impairment was recapitulated *in vitro* with the inability of SCs to divide, differentiate and self-renew in an environment containing serum from a septic mouse. Three months post induction of sepsis, the muscle was essentially unable to regenerate, which suggests a lasting impairment that resembles the impairment observed in the clinic and in patients that suffer from muscle force loss for up to 5 years post sepsis^45^,^46^.

We showed that the functionality of SCs was restored by mesenchymal stem cell injection^47^,^48^,^49^,^50^,^51^, leading to more efficient muscle regeneration, even when the treatment was performed during sepsis. Although the systemic decrease of cytokines is probably a part of the global mechanism leading to better regeneration of muscle during sepsis, our data do not exclude a more direct cell-to-cell rescue mechanism. The inability of the contralateral (non-injected) TA muscle to regenerate when the other TA muscle was treated with MSC suggests that local or direct MSC contact with SCs is required for rescue. The mitochondrial impairment in satellite cells occurs as early as 6 h post induction of sepsis, and MSC injection during this critical time promotes a rapid rescue of all mitochondrial and metabolic parameters. The rapidity of mitochondrial, and thereby cellular, impairment post sepsis as well as the recovery after MSC treatment suggests that critical parameters linked to the long-term effects are altered and can be rescued shortly after induction of sepsis. In this context, mitochondrial and metabolic parameters may act as read-outs of the status of critical cells during sepsis and recovery.

These findings are clinically relevant as sepsis is the major cause of critical illness myopathy, and administration of mesenchymal stem cells is a therapeutic approach currently tested in various acute diseases. Our findings provide evidence that stem cells are deeply affected by sepsis, but for this phenotype to emerge, SCs must be activated. In patients, such activation could occur years after sepsis, on stimulation of long-lasting quiescent SCs. The impaired regenerative potential of activated SCs can thereby explain, at least in part, long-term effects, notably muscle weakness, observed from several months up to 5 years after sepsis.

## Methods

### Mouse experiments

We used a peritonitis induced by CLP as a model of sepsis in mice C57Bl/6RJ from Charles River Laboratories or in-house transgenic *Tg:Pax7nGFP*^16^. For isolation of MSC we used either in-house transgenic *Tg:CAG-hPLAP::MLC3F-nlacZ-2* or *C57Bl/6Rj* mice from Charles River. All mice used in the study were male between 6 and 12 weeks old. Animals were anaesthetized with ketamine (Imalgene1000, 100 mg kg^−1^; Merial) and Xylazine (Rompun 2%, 20 mg kg^−1^; Bayer) before surgery. After laparotomy, caecum was ligatured at the distal third with a 4.0 suture and the distal part was perforated twice with a 21-Gauge needle. Animals were hydrated and treated with analgesic (buprenorphine, Axience, 0.3 mg kg^−1^) twice a day for 4 days following surgery. For injury mice were anaesthetized as previously described and 10 μl of 12.5 μg ml^−1^ notexin (Latoxan) was injected in the TA. All protocols were reviewed by the Institut Pasteur, the competent authority, for compliance with the French and European regulations on Animal Welfare and with Public Health Service recommendations. This project has been reviewed and approved (# 2013-0044) by the Institut Pasteur ethic committee (C2EA 89—CETEA).

### Thymidine analogue and hypoxyprobe injections

Cell proliferation was assessed using the thymidine analogue EdU incorporation. Animals received intraperitoneal injections of 5-ethynyl-2'-deoxyuridine (EdU Invitrogen#A10044) at 30 mg/kg 12 and 2 h before sacrifice. EdU was revealed using click-it reaction kit.

Hypoxia was detected using hypoxyprobe. Pimonidazole was injected at 120 mg/kg 90 min before sacrifice and revealed using monoclonal mouse antibody (Hypoxyprobe #HP1-1000) directed against pimonidazole and Goat Anti-Mouse IgG (H+L) Cy3 (ImmunoJackson #115-166-146).

### Histological analysis

TA was carefully dissected and snap frozen in liquid-nitrogen-cooled isopentane for 5 min and stored at −80 °C before cryosectioning (10-μm sections). Sections were kept at room temperature overnight before staining. Sections were then rehydrated in PBS for 10 min and fixed in 10% formalin for 3 min. The sections were then routinely stained with haematoxylin and eosin or with Sirius Red.

### Immunostaining

Immunostaining was performed on cryosections fixed with 4% paraformaldehyde (PFA; EMS#15710) in cold PBS, permeabilized with 0.5% Triton X-100 for 20 min at room temperature, washed and blocked with 10% BSA for 30 min. Sections were incubated with primary antibodies overnight at 4 °C and with Alexa-conjugated secondary antibodies 1/250 and Hoechst for 45 min (Supplementary Table 2). Sections were then analysed using an automated axioscan (Zeiss) or inverted Observer.Z1 Apotome (Zeiss). For apoptosis assessment (TUNEL assay and cleaved caspase-3 immunostaining), cells were collected in 2% serum, spun on poly-D-lysine (Sigma-Aldrich #P6407) and immediately fixed with 4% PFA . For mitochondrial immunostaining, freshly isolated adult muscle satellite cells were fixed with 2% PFA and permeabilized with 0.5% Triton X-100. Cells were incubated overnight in blocking buffer (BSA 5% in PBS) at 4 °C. Primary antibody was then added for 1 h at room temperature, followed by secondary conjugated antibody and 10 μg ml^−1^ Hoechst 33342 for 1 h at room temperature (Supplementary Table 2).

### Muscle cell sorting and culture

Muscle dissection was done by removing all of the limb muscle from the mice, in cold DMEM. Muscles were then chopped with small scissors and put in a 50-ml Falcon tube with collagenase 0.1% and trypsin 0.1% at 37 °C with gentle agitation. After 20 min, the supernatant was collected in 2% serum placed on ice, and the collagenase/trypsin solution was added to continue the digestion. Once muscle was completely digested, the solution was filtrated using 40-μm cell strainers. Satellite cells were cultured in 1:1 DMEM Glutamax (Gibco #41965-039):MCDB201 (Sigma #M6770) containing 20% serum FBS (Biowest S1860). When was used septic serum from mice, the serum was collected from 24 h post CLP septic mice serum extracted from blood sampled from the heart, spun at 400*g* at 4 °C. The supernatant was collected and the serum was kept frozen and thawed when preparing the medium. Medium was filtered using 0.22-μm filters. Cells were plated on Matrigel coating (BD Biosciences #354234) and kept in an incubator (37 °C, 5% CO_2_) at an initial concentration of 15,000 cells per mm^2^. For some *in vitro* experiments, serum was extracted from septic animals 24 h post CLP by heart puncture followed by centrifugation at 500*g* for 15 min. The thus-obtained supernatant replaced FBS in the culture medium; the rest of the medium was unchanged.

For satellite cell counting, only the TA muscle was dissected and digested as described earlier, and the totality of the tube was analysed to assess the number of satellite cells per muscle. FACS analyses were done using a FACSAria (Beckman). Analyses and quantitation were performed using Summit v4.3 software from DakoCytomation, and FloJo software. Cells were labelled with propidium iodide 10 μg ml^−1^ (Sigma-Aldrich #P4170) to exclude dead cells and displayed using the phycoerythrin (red) channel on the FACS profile.

### Isolation and culture of mesenchymal stem cells

MSCs were collected, cultured and characterized from C57BL/6J (6 to 8 weeks old) or *Tg:CAG-hPLAP::MLC3F-nlacZ-2E* mice. Briefly, in anaesthetized mice (injected intraperitoneally with 100 mg per kg body weight of ketamine and 5 mg per kg body weight of xylazine), femurs were flushed to recover bone marrow. For mesenchymal stem cells isolation, the cell suspension was filtered before red blood cell lysis and incubated with the following antibodies: allophycocyanin-conjugated PDGFR-α, FITC-conjugated Sca-1, phycoerythrin-conjugated CD45 and Ter119. Appropriate gates were constructed on a cell sorter to exclude dead cells and lineage (CD45(+)Ter-119(+))-positive cells. Cells were plated in tissue culture flasks, and cultured in 1 ml of complete medium at a density of 25 × 10^6^ cells per ml. Cells were incubated in plates at 37 °C with 5% CO_2_ in a humidified chamber. After 3 h, the supernatant was removed and non-adherent cells that accumulate on the surface of the dish were replaced by changing the medium. After an additional 8 h of culture, the medium was replaced with 1.5 ml of fresh complete medium. Thereafter these steps were repeated every 8 h for up to 72 h from the initial culture. After 2 weeks from the initiating culture, cells were washed in PBS and lifted by incubation in 0.5 ml of 0.25% trypsin/1 mM ethylenediaminetetraacetic acid (EDTA) for 2 min at room temperature.

### Live video microscopy

Cells isolated by FACS were plated overnight on a 24-well glass bottom plate (P24G-0-10-F; MatTek) coated with Matrigel (BD Biosciences #354234) and placed in an incubator in pre-equilibrated medium (1:1 DMEM Glutamax:MCDB (Sigma-Aldrich), 20% fetal calf serum (FCS; Biowest S1860). The plate was then incubated at 37 °C, 5% CO_2_ (Zeiss, Pecon). A Zeiss Observer.Z1 connected with a LCI PlnN × 10/0.8 W phase II objective and AxioCam camera piloted with AxioVision was used. Cells were filmed for up to 5 days, and images were taken every 30 min with bright-field and phase filters and MozaiX 3X3 (Zeiss). Raw data were transformed and presented as a video.

### Image analysis

For image analysis (fibrosis quantification) the ImageJ 1.46r software analysed 10 randomly taken photos per section and minimum 3 sections per experimental group. We converted each picture in a binary image and then collected the pixel values. For fibre size, the sections were immunostained with rabbit anti Laminin (Sigma-Aldrich #L9393) diluted at 1/200, overnight at 4 °C. Secondary Donkey anti-Rabbit Alexa-488 (DL488 JacksonImmuno #711486152) was used at 1/200 for 45 min at room temperature. The fibre perimeter was automatically evaluated using the Pixcavator software.

### Luminex

Snap-frozen TA samples (*n*=4 per condition) were thawed and lysed, and supernatant was processed for Luminex multiple cytokine and chemokine analysis (Bio-Plex ProTM Mouse Cytokine Standard 23-Plex, Group I; and Standard 9-Plex, Group II). Normalization was done by sample weight of frozen muscle. The study of inflammatory cytokines was conducted on control, septic and septic mice injected with MSCs.

### Mitochondrial DNA analysis

Total genomic DNA samples were subjected to PCR amplification with LA Taq DNA polymerase (Takara) to amplify two large mtDNA fragments that together span the whole mtDNA genome. PCR products were subjected to 0.8% agarose electrophoresis gel. Two sets of primers were used: forward 5′-GGAGCCTCAATATTTTTT-3′ and reverse 5′-AATGATGGCTACAACGAT-3′, to amplify the 9,898-bp fragment (coordinates 14,400–7,998 of the 16,299-bp-long *Mus musculus* mitochondrial genome NC_006914); and forward 5′-GATGAACAGTCTACCCAC-3′ and reverse 5′-GGGAGTAGCTCCTTCTTC-3′ to amplify the 9,677-bp fragment (coordinates 5,701–15,377).

### Mitochondrial Transcription and Replication Imaging Protocol

DNA probes for the fluorescence *in situ* hybridization protocol called Mitochondrial Transcription and Replication Imaging Protocol (mTRIP)^28^ was designed for the mouse mitochondrial genome and amplified using total mice DNA and the following sets of primers:

mREP (coordinates on the mouse mitochondrial DNA: 15655–15758), 103 bp: forward 5′-ATATGACTATCCCCTTCCCC-3′; reverse 5′-AGTTTAATGGGCCCGGAGCG-3′. mTRANS: equal mix 1:1:1 of 3 DNA probes MT1, MT6 and MT11. MT1 (1–1,021), 1,020 bp: forward 5′-GTTAATGTAGCTTAATAA-3′; reverse 5′-TTCCAAGCACACTTTCCA-3′. MT6 (7,021–7,998), 978 bp: forward 5′-CCCATTCCAACTTGGTCT-3′; reverse 5′-AATGATGGCTACAACGAT-3′. MT11 (13,140–14,357), 1,218 bp: forward 5′-GTCATCTCATATAATATT-3′; reverse 5′-TCGACAAATGTGTGTTAC-3′.

A unit of 1 μg of mREP and 1 μg of mTRANS mix were labelled by nick translation of PCR products, and incorporated in Atto550-dUTP following the manufacturer's procedure (Atto550 NT Labeling Kit, Jena Bioscience). For mREP or mTRANS staining, 40 ng of labelled probe were mixed with 400 ng of sonicated salmon sperm DNA (Life Technologies) in a final 25-μl volume of hybridization buffer (50% formamide, 10% dextran sulfate, in 2 × saline sodium citrate buffer (SSC; pH 7.0). DNA was denatured at 80 °C for 10 min and then kept at 37 °C for 30 min. Freshly isolated adult muscle stem cells were fixed with 2% PFA, permeabilized with 0.5% Triton X-100, incubated in 50% formamide/2 × SSC (pH 7.0) for 30 min at room temperature and denatured in 70% formamide/2 × SSC for 4 min at 75 °C. Hybridization was performed with 40 ng of probe for 16 h at 37 °C. Slides were washed in SSC, and DNA was stained with 10 μg ml^−1^ of Hoechst 33342.

### Carbonylated and nitrated protein levels

Freshly isolated adult muscle stem cells were fixed in 2% PFA and permeabilized with 0.5% Triton X-100. Carbonylated protein levels were measured with 0.01% (100 μg ml^−1^) dinitrophenyl derivatization in 2N HCl for 1 h at room temperature in the dark, followed by an overnight blocking step (5% BSA in PBS) at 4 °C. Polyclonal rabbit anti-dinitrophenyl antibody was then added for 1 h and secondary anti-rabbit antibody Alexa Fluor 555 Conjugate and with 10 μg ml^−1^ Hoechst 33342 for 1 h. Nitrated protein levels were measured by blocking permeabilized cells (5% BSA in PBS) overnight at 4 °C, followed by 1 h incubation with the primary anti-nitrotyrosine antibody, and 1 h with the secondary anti-rabbit antibody Alexa Fluor 555 Conjugate, and 10 μg ml^−1^ Hoechst 33342.

### Mitochondrial and ROS assays

Mitochondrial membrane potential was measured over 1 h on live, freshly isolated adult muscle stem cells using 200 nM tetramethylrhodamine ethylamine (Sigma-Aldrich). Cells were incubated for 1 h with the MitoTracker Deep Red staining (FM 8778S from Cell Signaling), a dye that stains mitochondria in live cells. ROS were measured by incubating CellRox (Life Technologies #C10422) 30 min at 37 °C and analysed by FACS.

### Preparation and immunostaining of single muscle fibres

The extensor digitorum longus muscles were dissected from tendon to tendon and treated for 1 h with 0.1% collagenase (C-0130, Sigma) at 37 °C. The muscles were then flushed to separate single fibres. Fibres were then transferred one by one into a dish using serum-coated Pasteur pipettes. Single fibres were then put in 20% FCS (Invitrogen) in DMEM (Invitrogen):MCDB201 (Sigma) (1:1) medium with penicillin and streptomycin, and fixed with 2% PFA in PBS at room temperature. When indicated, isolated muscle fibres were incubated for 1 h with MitoTracker Deep Red and then fixed in 2% PFA. Single fibres were immunostained for protein carbonylation, protein nitration and mitochondrial TOM22, following the respective procedures indicated above; immunostaining was performed in a 100-μl volume in 2-ml Eppendorf tubes. Confocal acquisitions were performed using a spinning-disk Perkin-Elmer Ultraview RS Nipkow Disk, an inverted laser-scanning confocal microscope Zeiss Axiovert 200M with an Apochromat × 25 objective and a Hamamatsu ORCA II ER camera (Imagopole, PFID, Institut Pasteur). Optical slices were taken at 200-nm intervals along the *z* axis covering the whole depth of the cell, at a 1.024/1.024 pixel resolution. Three-dimensional reconstruction was achieved using the IMARIS software (Bitplane). Fluorescence quantification per surface area was done using a single-imaging frame collection and ImageJ 1.47-v software (post-acquisition analysis).

### SOD activity

SOD activity was measured with Superoxide Dismutase Activity Colorimetric Assay kit (Abcam, ab65354), following the manufacturer's instructions. This measurement assesses SOD-dependent inhibition of superoxide anion reduction. Tests were performed with 10 μg of proteins, quantified by Bradford assay, from 50,000 cells, per sample.

### ATP assay

ATP levels were measured for 10,000 freshly isolated adult muscle stem cells using the CellTiter-Glo luminescent assay following the manufacturer's procedure (Promega). When indicated, 10 μM oligomycin (Sigma-Aldrich) was added for 1 h to inhibit mitochondrial ATP synthesis and assess glycolysis contribution.

### Cellular bioenergetics

Cellular bioenergetics was analysed on a Seahorse extracellular flux analyser (XFe96) according to the manufacturer's instructions. Briefly, freshly FACS-isolated SCs were seeded at a density of 200 × 10^3^ cells per well in a Seahorse XF96 plate and incubated for 3 h in 3%O_2_/5% CO_2_ at 37 °C. The seeding density and incubation conditions were based on initial assays that optimized oxygen consumption rate (OCR) and were compatible with attachment and survival of SCs (from control and sepsis mice). No OCR signal was indeed detected if the assay was performed immediately after isolation or 1 h incubation in 20%O_2_/5% CO_2_ at 37 °C, including in experiments performed in the presence of the cell immobilizer BD Cell Tak coating (BD Biosciences). Moreover, 3-h recovery in 3%O_2_/5% CO_2_ at 37 °C optimized the OCR signal. Immediately before the assay, cultured cells were washed twice in minimal assay media (Seahorse Biosciences, 37 °C, pH 7.40) supplemented with 25 mM glucose, 1 mM sodium pyruvate and 2 mM L-glutamine, then equilibrated for 1 h in a non-CO_2_ incubator. The basal rate of OCR and extracellular-acidification rate (ECAR), as well as OCR and ECAR in the presence of, successively, olygomycin (1 μM) that inhibits ATP synthase and distinguishes the part of mitochondrial respiration coupled to ATP production from that due to proton leak, FCCP (0.5 μM) that uncouples mitochondrial OXPHOS and measures maximal respiration and spare respiration capacity or cell ability to respond to increased energy demand, and antimycin A/rotenone (1 μM) that shut down mitochondrial respiration and measures non-mitochondrial respiration, were measured in *n*=3 mice per condition for 4 min (over a total of 24 min) from the rate of decline in O_2_ partial pressure (OCR), and rate of change in assay pH (ECAR).

### Three-dimensional confocal imaging and quantification

Confocal acquisitions were performed using a spinning-disk Perkin-Elmer Ultraview RS Nipkow Disk, an inverted laser-scanning confocal microscope Zeiss Axiovert 200M with an Apochromat × 100/1.4 oil objective and a Hamamatsu ORCA II ER camera (Imagopole, PFID, Institut Pasteur). Optical slices were taken at 200-nm intervals along the *z* axis covering the whole depth of the cell, at a 1.024/1.024 pixel resolution. Three-dimensional reconstruction was achieved using the IMARIS software (Bitplane). Fluorescence quantification was done using a single-imaging frame collection and ImageJ 1.47-v software (post-acquisition analysis).

### RT–qPCR

Total RNA was isolated from cells using the RNAeasy Micro kit (Qiagen), and reverse transcribed using Superscript III Reverse transcriptase (Invitrogen). RT–qPCR was performed using Power Sybr Green PCR Master Mix (Applied Biosystems) and the rate of dye incorporation was monitored using the StepOne Plus RealTime PCR system (Applied Biosystems). Three biological replicates were used for each condition. Data were analysed by StepOne Plus RT PCR software v2.1 and Microsoft excel. TBP transcript levels were used for normalization of each target (=ΔC_T_). RT–qPCR C_T_ values were analysed using the 
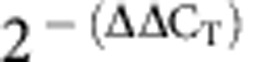
 method to calculate the fold expression.

### Quantification of mtDNA content by qPCR

Total DNA was prepared using extraction buffer (0.2 mg ml^−1^ proteinase K, 0.2% SDS and 5 mM EDTA in PBS) and incubated at 50 °C for 3 h. DNA was precipitated with 3 M sodium acetate (pH 5.2) and isopropanol for 20 min on ice before centrifugation at 8,000*g* at 4 °C. The DNA pellet was washed and air dried. qPCR amplification was performed on 200 pg of total DNA using the StepOne Plus RealTime PCR system (Applied Biosystems) and Power Sybr Green PCR Master mix (ABI) following the manufacturer's instruction. A fragment of mitochondrial *COI* gene, the established marker for mtDNA content in mouse cells, was amplified using the nuclear encoded Ndufv1 gene as endogenous reference. The level of mtDNA was calculated using the ΔC_T_ of average C_T_ of mtDNA and nDNA (ΔC_T_=C_T_ nDNA−C_T_ mtDNA) as 
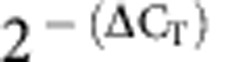
.

### *In vivo* perfusion imaging and phosphorus spectroscopy

All *in vivo* NMR experiments were performed at 4 T in a 20-cm free bore magnet (Magnex, Scientific Ltd) with a Bruker Biospec spectrometer (Bruker Medical Gmbh). Mice were positioned supine on a water-heating pad to ensure a stable body temperature at 37 °C. Animal anaesthesia was induced with 4% isoflurane (Forene, Abbott) and maintained with 1−1.75% isoflurane delivered in 1.5 l min^−1^ oxygen flow to keep a stable respiratory frequency.

Perfusion data were collected with a 1H whole-body 9-cm inner diameter transmitter volume coil for blood selective (*M*_+_) and non-selective (*M*_−_) tagging and a 2-cm diameter 1H surface coil for reception. The tissue blood flow was measured by a custom-developed STIR sequence (TR=10 s; *T*_ev_=1.2 s; NR=60), and gastrocnemius muscle blood perfusion (*f*; ml min^−1^ per 100 g) was extracted from perfusion maps as follows:


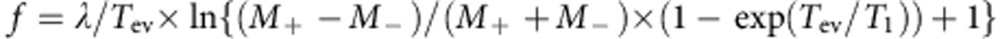


For calculations, the blood/tissue partition coefficient *λ* was fixed at 0.9 and muscle relaxation time *T*_1_ at 1.3 s.

Muscle metabolites were probed by a ^31^P saddle-shaped coil (10 mm) placed around the calf. After localized adjustment of field homogeneity on the water signal, ^31^P spectra were acquired at a repetition time of 2.5 s (100 μs broad pulse, spectral width 12 kHz, 4,096 data points, 360 NS (number of scans). Zero-filling (8 kHz), 8-Hz line broadening exponential multiplication, Fourier transform, manual phase correction and baseline correction were carried out on the acquired ^31^P spectra. Peak integrations were performed to calculate phosphorus metabolite ratios and pH:


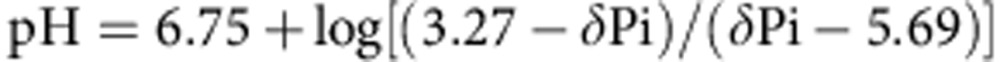


with *δ*Pi the chemical shift between phosphocreatine (PCr) and inorganic phosphate (Pi).

### *In vivo* force measurements

The grip strength test is a non-invasive method designed to evaluate mouse muscle force *in vivo.* A grip meter (Bio-GT3, BIOSEB), attached to a force transducer, measured the peak force generated. Septic and MSC-injected (21 days post injury) mice were placed with the four paws on a grid and gently pulled backward until they released the grip. Five trials were conducted for hindlimb grip strengths and, before statistical analysis, a mean value was calculated for each mouse using the tree median data. Results are expressed as the result of tree peak forces (in g), normalized to the body weight (in g).

### Skinned fibre experiments

TA muscles were dissected from septic and MSC-injected (21 days post injury) mice. Small bundles of two to five fibres were manually isolated from the muscles as previously described. Chemical skinning was carried out using Triton X-100. Skinned fibres were mounted in the Displacement Measuring System KD 2300 (model 0.5 SU, Kaman Instrumentation, Colorado Springs, CO, USA) To perform force measurements. Skinned fibre preparations were incubated for 1 h in relaxing solution (pCa 9.0, low calcium content) (pCa=−log_10_[Ca^2+^]) containing 1% Triton X-100 (v/v) to solubilize the sarcolemma and the sarcoplasmic reticulum membranes, and were subsequently washed several times in relaxing solution without detergent. Fibres were adjusted to slack length and then stretched progressively until the tension developed at pCa 4.5 (activating solution, high calcium content) became maximal. Tension/pCa relationships were obtained by exposing skinned fibres sequentially to solutions of decreasing pCa until maximal tension (*T*_max_) was reached (at pCa 4.5). Isometric tension was recorded continuously using a chart recorder (model 1200, Linear, Reno, Nevada, USA). To obtain Ca^2+^ sensitivity (pCa_50_) and Hill coefficient values (*n*_H_), data for the relative tension (*T*/*T*_max_) were fitted by using a modified Hill equation^41^. The tension obtained at each [Ca^2+^] was normalized to fibre cross-sectional area. The relaxing (pCa=9.0) and activating (pCa=4.5) solution compositions were calculated according to Godt and Nosek^42^. Solutions with intermediate [Ca^2+^] were obtained by mixing the pCa 9.0 and pCa 4.5 solutions in appropriate proportions.

### Statistical analysis

Statistical analysis was performed using GraphPad Prism software using appropriate tests (non-parametric Mann–Whitney unless specified) and a minimum of 95% confidence interval for significance; *P*-values indicated on figures are <0.05 (*), <0.01(**) and<0.001 (***). Figures display average values of all animals tested±s.d, or ±s.e.m. for RT–qPCR and qPCR, or as specifically indicated for the other experiments.

## Additional information

**How to cite this article:** Rocheteau, P. *et al.* Sepsis induces long-term metabolic and mitochondrial muscle stem cell dysfunction amenable by mesenchymal stem cell therapy. *Nat. Commun.* 6:10145 doi: 10.1038/ncomms10145 (2015).

## Supplementary Material

Supplementary Supplementary Figures 1-6 and Supplementary Tables 1-2

Supplementary Movie 1 Time-lapse of FACS cell sorted from control (healthy) Tg:Pax7nGFP mouse plated in healthy mouse serum.

Supplementary Movie 2 Time-lapse of FACS cell sorted from septic (CLP) Tg:Pax7nGFP mouse plated in healthy mouse serum.

Supplementary Movie 3 Time-lapse of FACS cell sorted control (healthy) Tg:Pax7nGFP mouse plated in septic mouse serum.

Supplementary Movie 4 Time-lapse of FACS cell sorted septic (CLP) Tg:Pax7nGFP mouse plated in septic mouse serum.
