# Supplementary material for: Sepsis induces long-term metabolic and mitochondrial muscle stem cell dysfunction amenable by mesenchymal stem cell therapy
Source: Nat Commun. 2015 Dec 15;6:10145. doi: 10.1038/ncomms10145 (PMC4682118; doi:10.1038/ncomms10145)
Supplement: Supplementary — Figures 1-6 and Supplementary Tables 1-2 [file ncomms10145-s1.pdf]

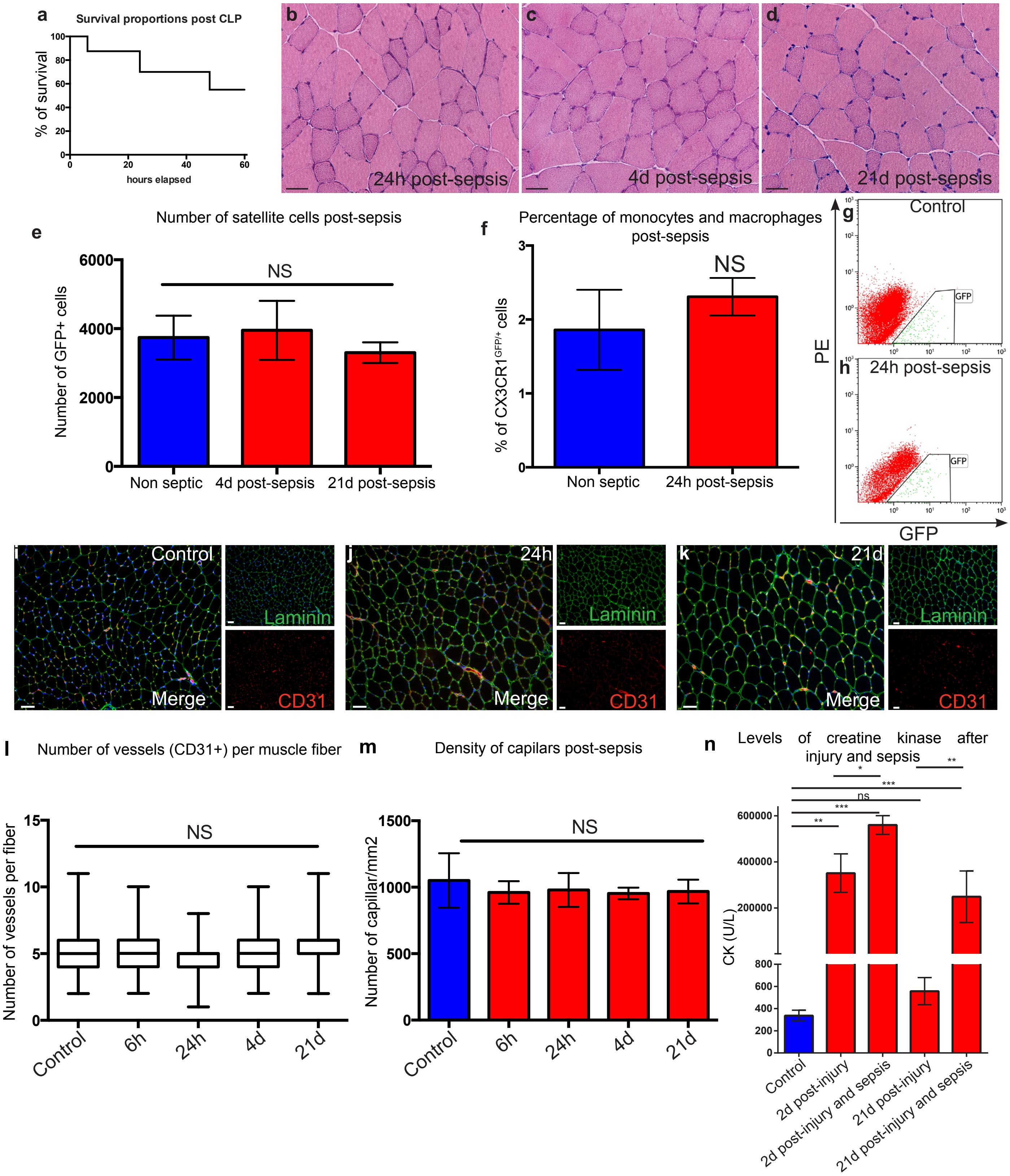

Supplementary. Fig. 1

Supplementary Fig. 1. Despite normal histology, muscle displays extensive hypoxia 24 hours post-induction of sepsis

(**a**) Survival curves of septic animals (n=8 per time point) (**b-d**) haematoxylin and eosin staining at 24h (**b**), 4d (**c**), 21d (**d**) post-sepsis. (**e**) Number of satellite cells counted by FACS in one tibialis anterior (TA) in control (non-injured, non-CLP), 4d and 21d post-CLP (without injury). (**f**) Histogram displaying the percentage of monocytes counted by cytometry in one digested TA of $CX3CR1^{GFP/+}$ mice. (**g-h**) Cytometric representation of $CX3CR1^{GFP/+}$ cells. (**g**) Control (n=5) (**h**) 24h post-sepsis (n=5). (**i-m**) Fibre size and vessel number in control and septic mice. (**i-k**) Immunofluorescence of Laminin (green); CD31 (red); and Dapi (bleu) merge and separated channels of (**i**) control; (**j**) 24h post-sepsis; (**k**) 21d post-sepsis. (**l**) Number of vessels per fiber in control, 6h, 24h, 4d, 21 days post-sepsis. (**m**) Density of capillary (absolute number per mm^2 during sepsis) in the control and 6h, 24h, 4d, 21 days post-sepsis. (**n**) Levels of creatine kinase measured in mouse plasma by colorimetric technique. Data are represented as mean ± s.e.m. $*P < 0.05$; $**P < 0.01$; $***P < 0.001$; ns, statistically non-significant, compared to the respective control. Scale bar: 100µm.

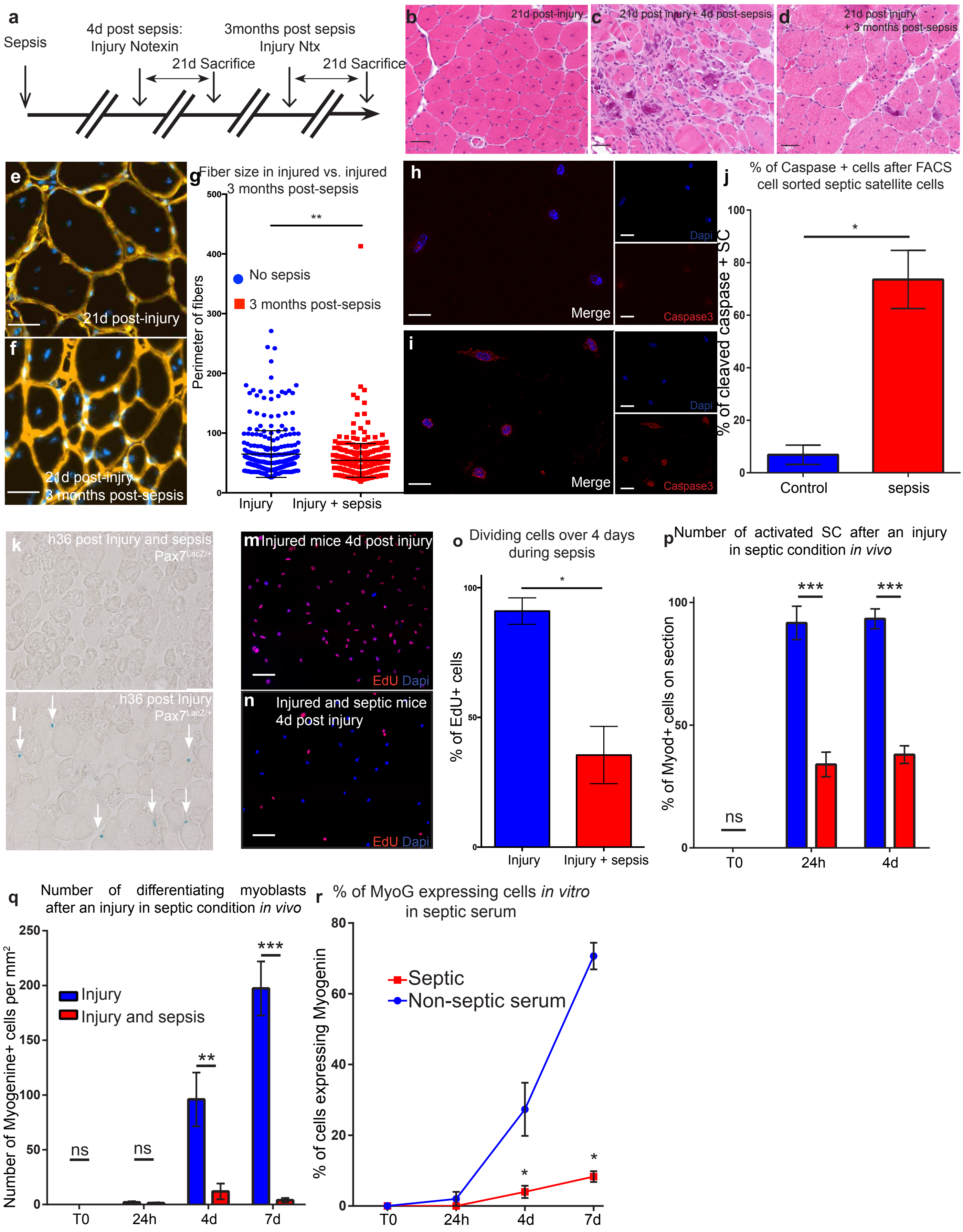

Supplementary. Fig. 2

Supplementary Fig. 2. Effects of sepsis on muscle regeneration last for extended periods of time

(**a**) Schematic representation of injury after extended periods of time post-induction of sepsis. (**b**) Haematoxylin eosin of a cryosetioned muscle 21 days post-injury of a control mouse C57Bl/6. (**c**) Haematoxylin eosin of a cryosetioned muscle from a C57Bl/6 mouse injured 4 days post-induction of sepsis. (**d**) Haematoxylin eosin of a cryosetioned muscle from a C57Bl/6 mouse injured 3 months post-induction of sepsis. (**e**) Laminin staining of 21 days post-injury muscle fromC57Bl/6 mouse (n=8 mice). (**f**) Laminin staining of 21 days post-injury muscle from C57Bl/6 mouse injured 3 months post-induction of sepsis (n=8 mice). (**g**) Perimeter of the fibres (in pixels) of a 21 days post-injury mouse and a 21 days post-injury mouse and injured 3 months post- induction of sepsis. (**h**) Cleaved caspase 3 immunostaining of FACS cell sorted SC 36h after injury of *Tg:Pax7nGFP* mouse. (**i**) Cleaved caspase 3 immunostaining of FACS cell sorted SC 36h after injury and septic of *Tg:Pax7nGFP* mouse. (**j**) Percentage of cleaved caspase 3 positive SC in control (36h post-injury) and sepsis (36h post-injury and sepsis). (**k**) 10µm cryosection of 36h post-injury and sepsis $Pax7^{LacZ/+}$ mouse after X-gal staining. (**l**) 10µm cryosection of 36h post-injury $Pax7^{LacZ/+}$ mouse after X-gal staining. (**m**) *Tg:Pax7nGFP* injured with notexin received twice a day a pulse of EdU at 50mg/Kg and were sacrificed 4 days post-injury (control) or (**n**) *Tg:Pax7nGFP* were injured with notexin, received a CLP to induce sepsis, received twice a day a pulse of EdU at 50mg/Kg and were sacrificed 4 days post-injury (septic). (**o**) Histogram representing the percentage of dividing cells over 3 days of injection of EdU *in vivo* in *Tg:Pax7nGFP* injured with notexin and sacrificed 4 days post-injury (injury) or *Tg:Pax7nGFP* injured with notexin, undergone sepsis, and sacrificed 4 days post-injury (injury + sepsis). (**p**) Percentage of SC Myod+ counted on section *in vivo* at T0 (right after the injury and induction of sepsis) 24h and 4 days after injury and sepsis. (**q**) Number of Myogenine+ cells per mm^2 counted on section *in vivo* after injury or injury and sepsis. (**r**) Percentage of Myogenine+ cells (among FACS cell sorted *Pax7-GFP* cells) *in vitro* in normal and septic mouse serum. Data are represented as mean ± s.e.m. $*P < 0.05$; $**P < 0.01$; $***P < 0.001$; ns, statistically non-significant, compared to the respective control. Scale bar: 100µm.

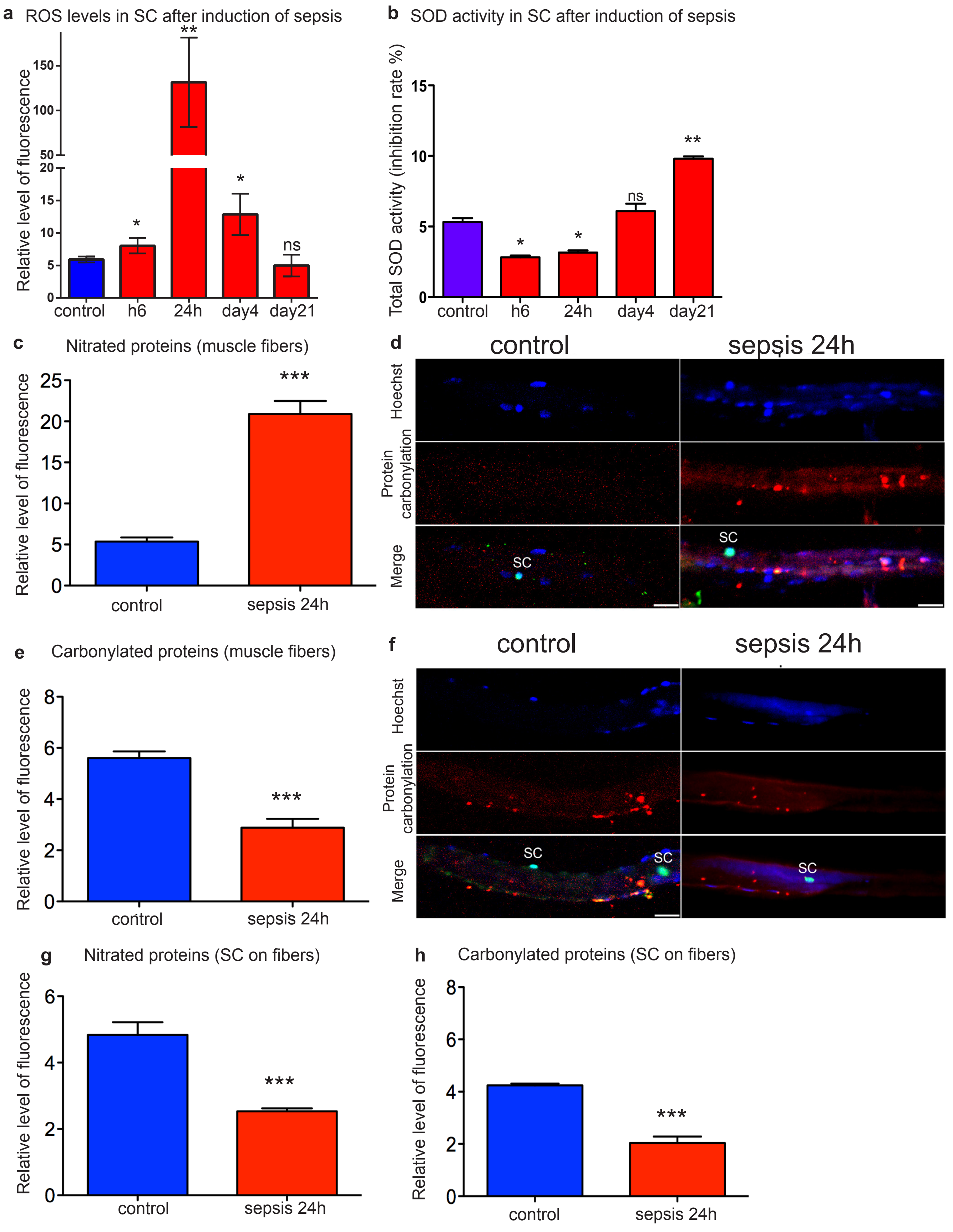

Supplementary. Fig. 3

Supplementary Fig 3. Oxidative and nitrosative stress in isolated SC and myofibers

(**a**) ROS levels measured by CellRox and (**b**) superoxide dismutase activity measured by colorimetric analysis in SC control and at different time points post-sepsis. Quantification of fluorescence intensity displaying the level of (**c**) carbonylated and (**e**) nitrated proteins in myofibers isolated from controls and 24h post-induction of sepsis, and (**d, f**) representative immunostaining (3D-reconstructed fibers). Pax7-GFP+ cells (SC) within the myofiber are indicated. Quantification of (**g**) carbonylated and (**h**) nitrated proteins in SC within the myofiber. (a,b) mean ±s.d, n=3 experiments; (c, e, g, h) mean ± s.e.m. *$P < 0.05$; **$P < 0.01$; ***$P < 0.001$; ns, statistically non-significant, compared to the respective control. Mann-Whitney test was used. Scale bar 10µm.

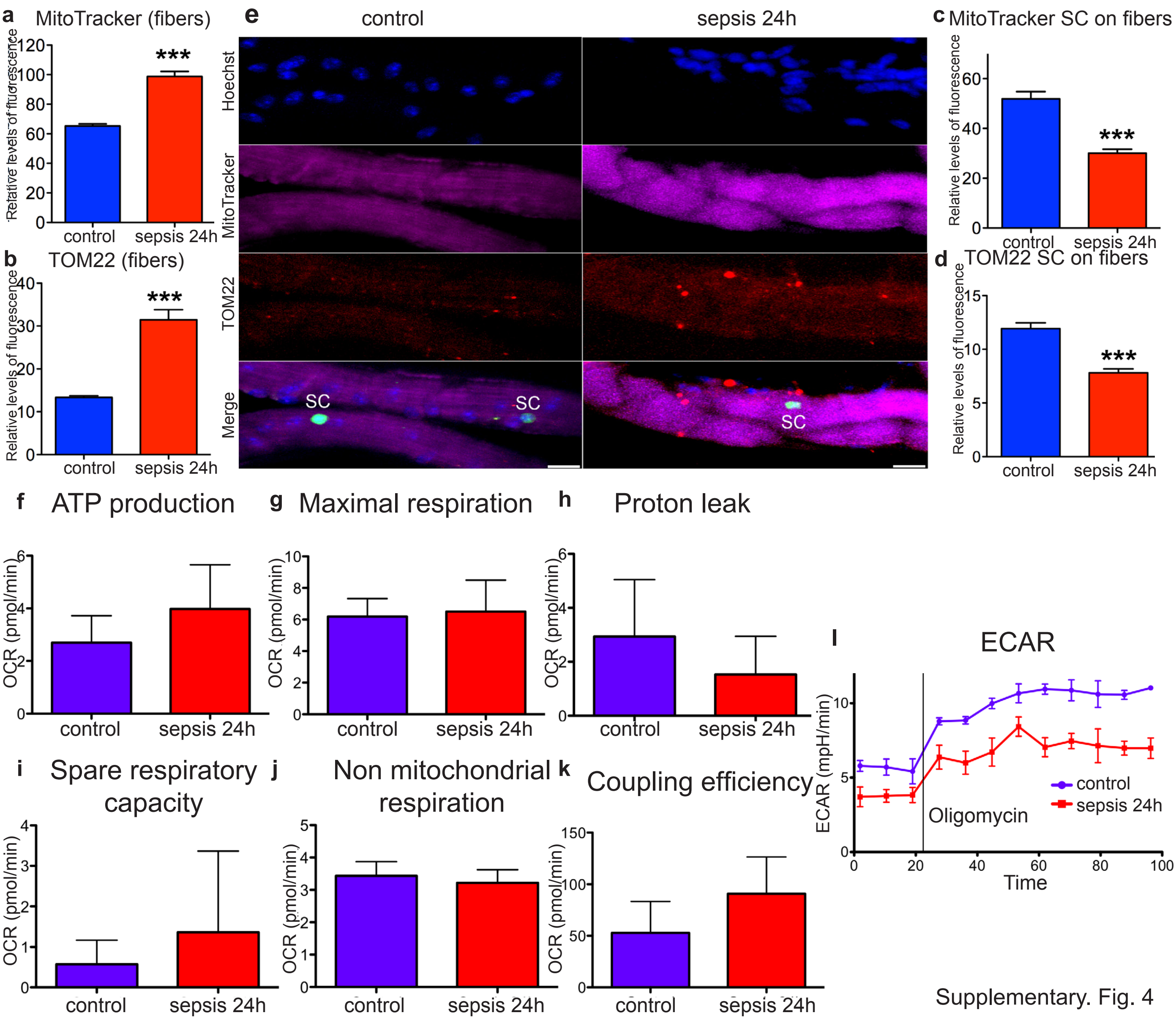

Supplementary. Fig. 4

Supplementary Fig 4. Mitochondrial mass in the myofiber and bioenergetics of SC

(**a**) Intensity of MitoTracker Deep Red staining and (**b**) fluorescence intensity of TOM22 immunostaining in myofibers, and SC within the myofiber (**c**, **d**) of control (healthy) and septic mice and (**e**) representative stainings (3D-reconstructed fibers). Pax7-GFP+ cells (SC) within the myofiber are indicated. Mann-Whitney test was used. Mean ± s.e.m. ***$P < 0.001$, compared to the respective control. Scale bare 10µm. (**f-i**) Cellular bioenergetics in freshly isolated SC form control and septic mice (24h post-sepsis) evaluated by Seahorse XFe96 analyzer. Critical bioenergetics parameters were calculated using the method described by Seahorse, and expressed as oxygen consumption rate (OCR) (**l**) Extracellular-acidification rate (ECAR), which reflects glycolysis activity, was measured for 4 min intervals (over a total of 24 min). Mean ±s.d, n=3 mice.

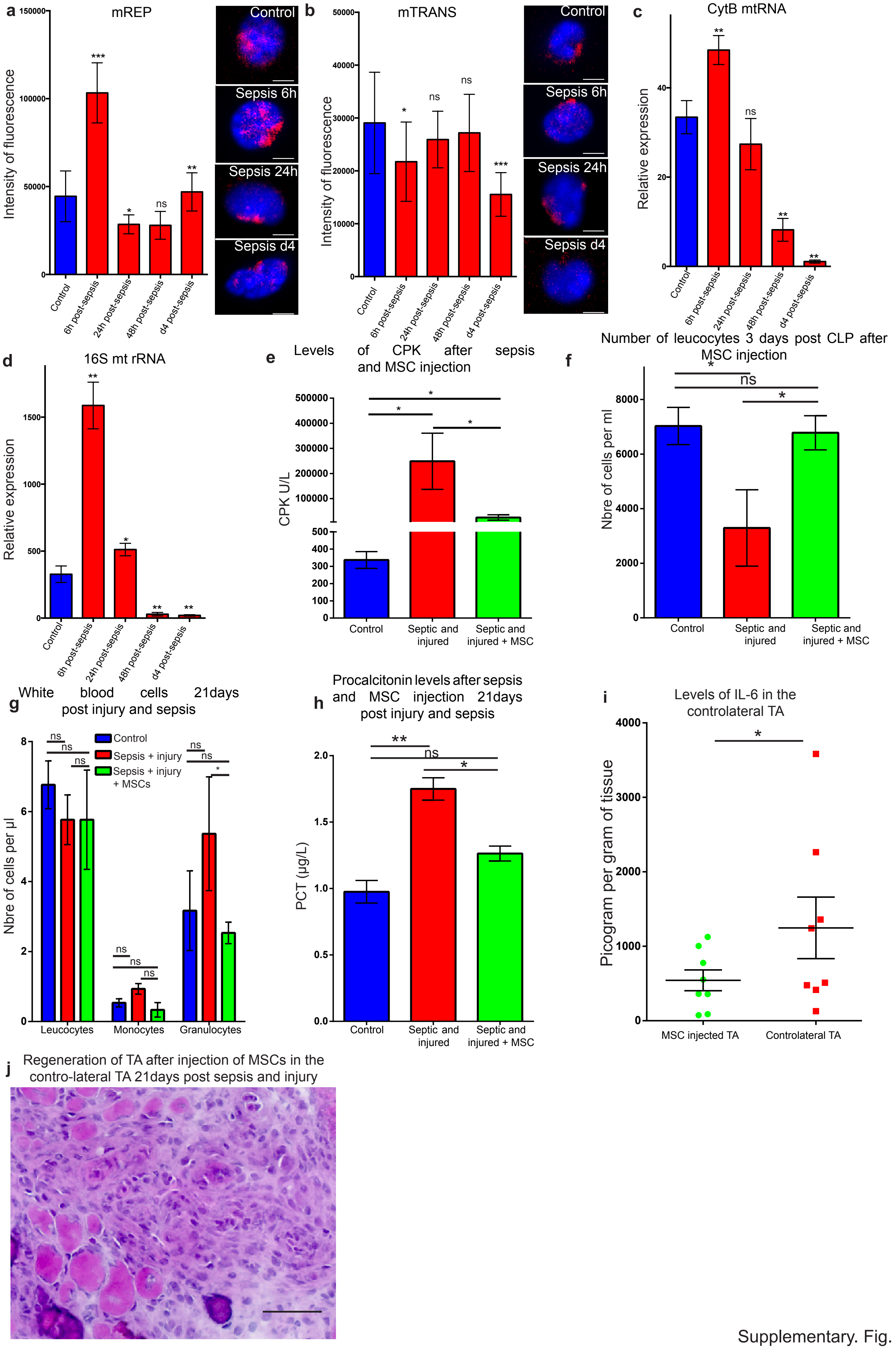

Supplementary. Fig. 5

Supplementary Fig. 5. Sepsis induces a state of hyper-activated mitochondria in SC at acute phase and restores inflammatory parameters 21 days post-sepsis and injury

(**a**) mREP (mitochondrial initiation of replication) signal at different time points post-induction of sepsis. *Right panels*, representative images of mREP-labelled SC. (**b**) mTRANS (global mitochondrial RNA) signal at different time points post-induction of sepsis. *Right panels*, representative images of mTRANS-labelled SC. (**c**) RT-qPCR of mitochondrial-coded *CytB* mRNA and (**d**) *16S* rRNA levels at different time points post-induction of sepsis. (**e**) Levels of Creatine phospho-kinase (CPK) in the plasma of control (no injury no sepsis), septic and injured, and septic and injured and MSCs injected mice. (**f**) Number of leucocytes in the blood 3 days after induction of CLP in the control (sepsis), septic and injured, septic and injured and MSC injected mice. (**g**) Number of white blood cells in control (injured only), septic and injured, and septic and injured and MSCs injected mice. Leucocytes, monocytes and granulocytes were automatically identified during the blood count. (**h**) Procalcitonin levels determined by ELISA in the plasma of mice in control (no sepsis, no injury), sepsis and injury, and sepsis and injury and MSCs injection 21 days post induction of sepsis and injury. (**i**) Levels of IL6 in septic and non-injected contralateral TAs. IL6 levels are displayed in picogram per gram of tissue in the septic injured and MSC injected TA (MSC injected TA), and in the septic injured and non-injected contralateral TA. (**j**) Hematoxylin eosin staining of an injured septic contralateral TA. When the left TA was injected with MSCs, the right TA did not regenerate in septic mice. Mann-Whitney test was used. Data are represented as mean ± s.d. *$P < 0.05$; **$P < 0.01$; ***$P < 0.001$; ns, statistically non-significant, compared to the respective control. Scale bar: 100µm

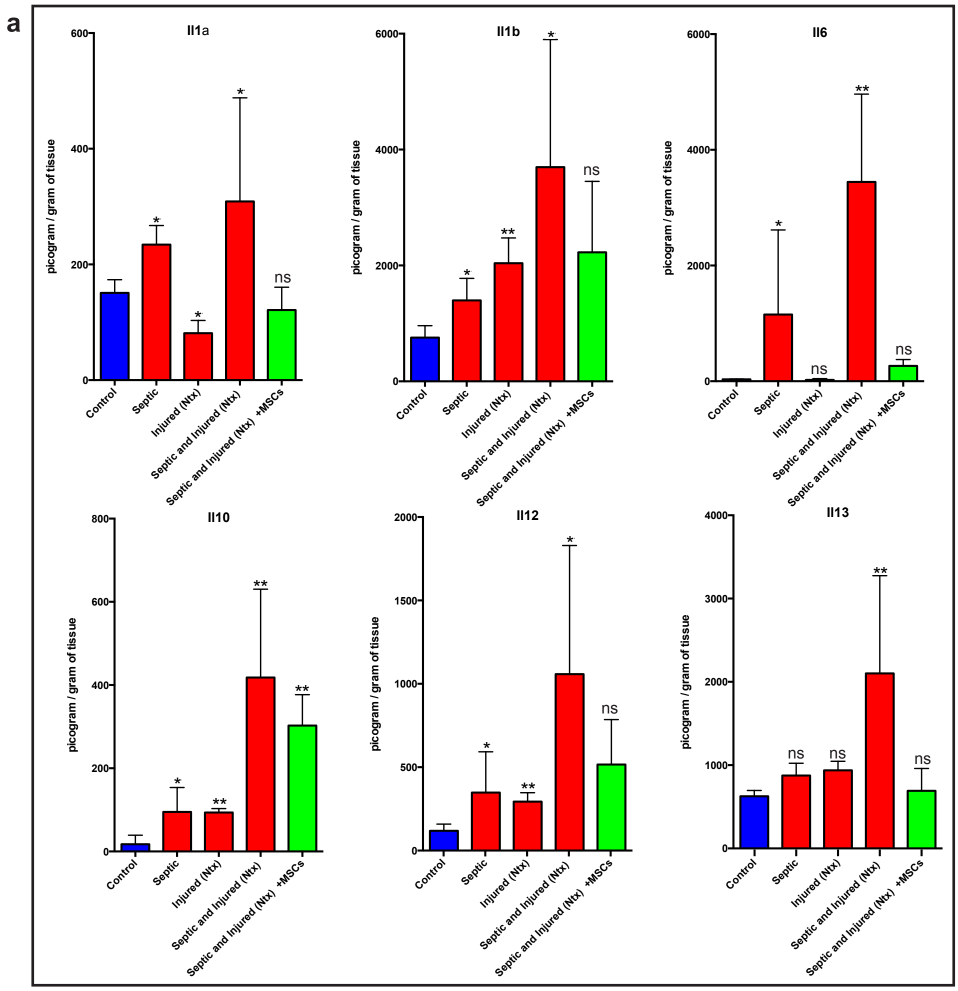

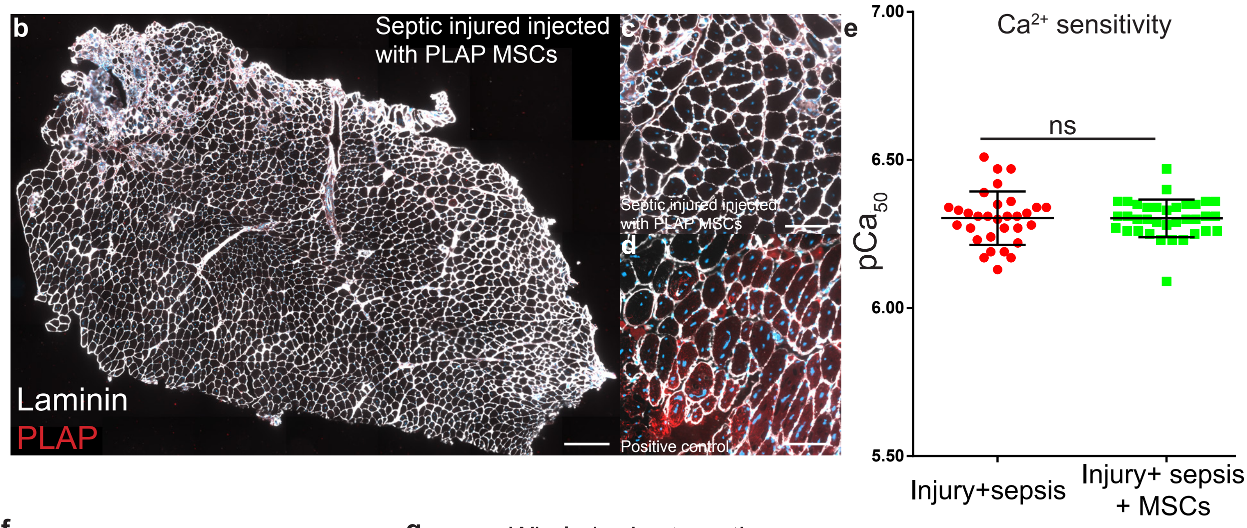

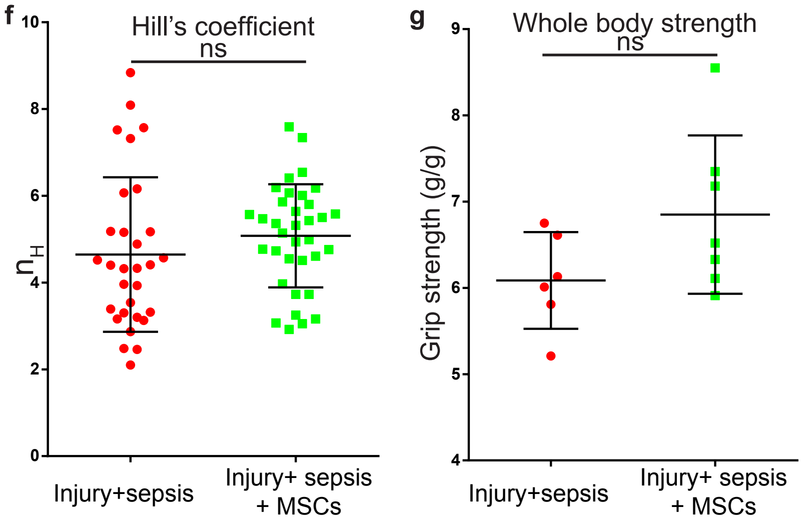

Supplementary. Fig. 6

Supplementary Fig. 6. MSC injection triggers a cytokines drop at the acute phase of sepsis and improves muscle force without transdifferentiation of MSCs.

(**a**) Luminex (multiplex assay) measuring the levels of cytokines in C57Bl/6 mice in pg of protein per gram of tissue in control; septic 24h; injured with notexin; septic 24h and injured with notexin; septic 24h injured with notexin and injected with mesenchymal stem cells. (**b**) Immunostaining of Laminin (white) and PLAP (red) of whole muscle section. Ten sections per animal were counted in septic, injured, and grafted animals (n=6 per condition). Scale bar represents 500µm. (**c**) Magnification of the immunostaining Laminin (white) and PLAP (red). Scale bar represents 100µm. (**d**) Positive control of PLAP positive satellite cells grafted to a C57Bl/6 mouse Laminin (white) and PLAP (red). (**e**) Ca^{2+} sensitivity of isolated fibers TritonX100 treated from injured and septic, or injured, septic and MSC injected C57Bl/6 mice. Data are represented mean±s.d. (**f**) Hill's coefficient of isolated fibers TritonX100 treated from injured and septic, or injured, septic and MSC injected C57Bl/6 mice. Data are represented mean±s.d (**g**) Whole body force (grip test) analysed on septic and injured (n=6) and septic and injured with MSCs injection (n=7). Unless specified data are represented as mean ± s.e.m. *P < 0.05; **P < 0.01; ***P < 0.001; ns, statistically non-significant, compared to the respective control. Scale bar: 100µm

Summary table of MNR results

Mice	Perfusion	PCr/ATPg	pH
CLPh24 1	3.92	1.68	6.65
CLPh24 2	8.81	2.42	7.04
CLPh24 3	5.94	2.34	7.12
CLPh24 4	40.22	1.84	6.86
CLPh24 5	15.17	2.40	7.05
CLPh24 6	12.03	2.82	7.18
CLPh24 7	6.25	2.79	6.99
CLPh24 8	4.27	3.60	7.21
CLPh24 9	1.33	1.17	6.74
**Mean CLPh24**	**10.88**	**2.34**	**6.98**
SD CLPh24	11.80	0.72	0.19

WTh24 1	8.83	ND	ND
WTh24 2	4.87	4.69	7.29
WTh24 3	6.24	3.25	7.20
WTh24 4	4.08	3.28	7.18
WTh24 5	7.83	3.05	7.10
WTh24 6	9.51	3.23	7.17
**Mean WT**	**6.89**	**3.50**	**7.19**
SD WTh24	2.18	0.67	0.06

Mann-Whitney WTh24 vs. CLPh24	0.97	0.018	0.041

Supplementary Table 1

Supplementary Table. 1. MNR measurements of septic mice.

For each mice, perfusion is indicated in ml/min/100g of tissue. Pcr/ATPg indicates the metabolic status of the tissue. Value of 9 septic (24h post-CLP) and 6 control C57Bl/6 mice, and their respective mean±s.d. are shown on the upper and the middle part of the table respectively. *P*-values of septic *vs.* control mice are shown in the lower part (Mann-Whitney test).

Antigen	Host	Concentration	References
Cleaved caspase-3 (Asp175)	Rabbit	5µg/ml	Cell Signaling (9661)
Myogenin (F5D)	Mouse	4µg/ml	Thermo scientific (MA5-11486)
GFP	Chicken	1µg/ml	Abcam (ab13970)
PLAP	Rabbit	4µg/ml	GeneTex (GTX73609)
CD31	Rat	15µg/ml	BD Pharmingen 550274
Pax7	Mouse	12µg/ml	DSHB
Laminin	Rabbit	0.69µg/ml	Sigma-Aldrich L9393
TOM22	Rabbit	0.69µg/ml	Sigma-Aldrich HPA003037
Nitrotyrosine	Rabbit	0.5µg/ml	Life Technologies A21285
Dinitrophenol	Rabbit	0.5µg/ml	Merck 57150
Pimonidazole	Mouse	1µg/ml	Hypoxyprobe Inc. HP1
Secondary Donkey anti Rabbit (IgG Fraction Monoclonal)	Variable according to the primary Ab host	0.5µg/ml	JacksonImmuno #711486152 (Rabbit) #200162037 (Mouse)

Supplementary Table 2: list of antibody used in the study

SupplementaryTable. 2. List of antibodies used in the study.

For each antibody the reference and the dilution is displayed.
